# Laser printed microelectronics

**DOI:** 10.1038/s41467-023-36722-7

**Published:** 2023-02-27

**Authors:** Liang Yang, Hongrong Hu, Alexander Scholz, Florian Feist, Gabriel Cadilha Marques, Steven Kraus, Niklas Maximilian Bojanowski, Eva Blasco, Christopher Barner-Kowollik, Jasmin Aghassi-Hagmann, Martin Wegener

**Affiliations:** 1 Institute of Nanotechnology (INT), Karlsruhe Institute of Technology (KIT), 76128 Karlsruhe, Germany; 2 Institute of Applied Physics (APH), Karlsruhe Institute of Technology (KIT), 76128 Karlsruhe, Germany; 3 Institut für Organische Chemie, Ruprecht-Karls-Universität Heidelberg, Im Neuenheimer Feld 270, 69120 Heidelberg, Germany; 4 Institute for Molecular Systems Engineering and Advanced Materials (IMSEAM), Ruprecht-Karls-Universität Heidelberg, Im Neuenheimer Feld 225 and 270, 69120 Heidelberg, Germany; 5 School of Chemistry and Physics, Queensland University of Technology (QUT), 2 George Street, Brisbane, QLD 4000 Australia; 6 Centre for Materials Science, Queensland University of Technology (QUT), 2 George Street, Brisbane, QLD 4000 Australia; 7 Present Address: Suzhou Institute for Advanced Research, University of Science and Technology of China (USTC), 215127 Suzhou, China

**Keywords:** Electronic devices, Laser material processing

## Abstract

Printed organic and inorganic electronics continue to be of large interest for sensors, bioelectronics, and security applications. Many printing techniques have been investigated, albeit often with typical minimum feature sizes in the tens of micrometer range and requiring post-processing procedures at elevated temperatures to enhance the performance of functional materials. Herein, we introduce laser printing with three different inks, for the semiconductor ZnO and the metals Pt and Ag, as a facile process for fabricating printed functional electronic devices with minimum feature sizes below 1 µm. The ZnO printing is based on laser-induced hydrothermal synthesis. Importantly, no sintering of any sort needs to be performed after laser printing for any of the three materials. To demonstrate the versatility of our approach, we show functional diodes, memristors, and a physically unclonable function based on a 6 × 6 memristor crossbar architecture. In addition, we realize functional transistors by combining laser printing and inkjet printing.

## Abstract (web summary)

Printed organic and inorganic electronics continue to be of large interest for several applications. Here, the authors propose laser printing as a facile process for fabricating printed electronics with minimum feature sizes below 1 µm and demonstrate functional diodes, memristors, and physically unclonable functions.

## Introduction

Throughout the last two decades, many different functional organic^1–3^ and inorganic^4–6^ electronic devices have been realized by inkjet printing^7–9^, laser-induced forward transfer (LIFT)^10,11^, or other printing modalities^12–15^, rather than by high-vacuum deposition of materials and standard cleanroom lithographic means. Frequently, however, some fabrication steps use technologies other than direct printing, such that the majority of published devices is not yet fully printed. Moreover, typical minimum feature sizes usually lie in the range of tens of micrometers^16^. Only for special materials under special optimized conditions, such as, electrically polarizable inks^17,18^, patterned substrates^19,20^, or nanogaps^21^, feature sizes in the range of 1 µm have been accomplished. Clearly, large minimum feature sizes lead to undesirably large overall sizes of circuits built along these lines and tends to limit the reachable operation speeds. Finally, most solution-processable functional materials so far require some sort of sintering process of the involved semiconductors and/or metals at elevated temperatures. This complicates the processing of multilayer devices because caution has to be exerted that the sintering of one material component does not deteriorate any of the other material components involved.

Laser printing based on two-photon (multi-photon)^22,23^ or two-step^24^ absorption has been introduced close to two decades ago, but has mostly been used for the making of micro- and nanostructures based on polymeric materials. In these processes, a liquid monomer is locally polymerized, hence solidified, triggered by the absorption of light by photoinitiator molecules within the laser focus. For immersion objective lenses with a high numerical aperture (NA = 1.4) and for visible light, the lateral full width at half maximum of the laser focus can be as small as 0.1 µm. After scanning the laser focus along a desired pattern, the insufficiently crosslinked, hence still liquid, regions are washed out in a development step. Only more recently, multi-photon multi-material laser printing has picked up speed. For details, we refer the reader to a recent corresponding comprehensive review article^25^. However, so far, the emphasis has been on optical, mechanical, and biological applications of multi-photon laser-printed multi-material architectures. No functional electronic devices have been realized so far. This shortcoming can be traced back to the lack of suitable inks allowing for laser printing of electronically active semiconductor materials that can be combined with metallic contacts and interconnects in the printing process.

Here, we introduce laser printing as a facile process for fabricating a variety of functional electronic devices composed of a semiconductor (ZnO) and metals (Pt and Ag). We achieve <1 µm minimum feature sizes. We emphasize that none of the completely laser-printed functional devices that we present in this article need any sort of sintering after the printing process. The developed materials and laser printing method provide a solid fundament for printed electronic devices, such as diodes and memristors. The first generation of our laser-printed memristors show an on/off ratio exceeding ${10}^{2}$ as well as high retention and endurance. These features and the possibility to directly write the metal interconnects make them suitable for integration in crossbar architectures, to build, for example, physically unclonable functions (PUFs) for identification, authentication or encryption purposes and harvest the stochastic nature of the memristive behavior. Our miniature security circuits could directly be laser printed onto products, without any prior chip tape-outs or intellectual property transfer to the manufacturer, which would strengthen the root of trust.

### Laser microprinting of Pt, Ag, and ZnO

**Fig. 1:**
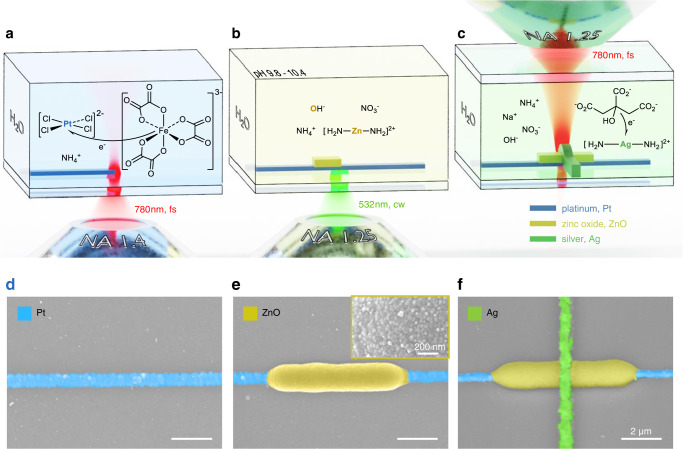
Direct laser printing of microelectronic structures. **a** Scheme of a femtosecond laser centered around 780 nm wavelength that is focused into the Pt ink for multi-photon reduction of Pt. **b** A continuous-wave laser at 532 nm wavelength is focused onto a Pt wire immersed in the ZnO ink for the local photothermal synthesis of ZnO. **c** The 780 nm femtosecond laser is focused above the ZnO layer within the Ag ink for further multi-photon reduction of Ag. **d**–**f** Scanning-electron micrographs of laser-printed structures corresponding to the process steps **a**–**c**, colored according to the different materials involved. The inset in **e** reveals the surface of a laser-printed ZnO structure.

Laser microprinting of platinum metal structures has recently been investigated in detail^26^ (also see ref. ^27^). The Pt-“ink” in this work consists of a Pt(II) salt and an iron(III) oxalate photosensitizer (also refer to “Methods”). The focused near-infrared femtosecond laser pulses lead to reduction of Pt-ions by multi-photon-absorption of the iron oxalate photosensitizer. The initially formed Pt nanoparticles are either ejected from the laser focus or are sintered together on the glass substrate surface by local heating, which is induced by one-photon absorption of the laser light by the platinum nanoparticles themselves (see Fig. 1a). In this process, light-induced forces onto the metal nanoparticles play a major role for nanoparticles aggregation as well^26^. Due to the large thermal conductivity of the surrounding aqueous ink, the heating can be strongly localized to below a micrometer^28^. The electrical conductivity of laser-printed Pt lines has been characterized to be ${\approx 2.4\times 10}^{5}$ S/m (${9.4\times 10}^{6}$ S/m for bulk platinum)^27^.

The Ag-“ink” in this work consists of a Ag(I) complex and a citrate reducing agent (see “Methods”). Here the photoreaction triggered by focused near-infrared femtosecond laser pulses proceeds via multi-photon-absorption of the Ag(I) complex followed by reduction of the excited state species. Laser printing of silver has also been discussed many times in the literature^29–32^ and is possible with laser-focus speeds up to $v=1\;{{{{{\rm{cm}}}}}}/{{{{{\rm{s}}}}}}$ for optimized inks^33^. Laser-printed polycrystalline silver metal lines tend to be grainier than platinum lines (see below). Notably, silver is a standard material in inkjet-printed electronics as well as for many types of memristors in general. Polycrystalline silver has a work function of 4.26 eV^34^, which complements the much larger work function of platinum of 5.64 eV^35^. The electrical conductivity of laser-printed Ag lines has been characterized to be ${\approx 5.9\times 10}^{6}$ S/m (${6.3\times 10}^{7}$ S/m for bulk silver)^29,33^. Gold can be laser printed along the same lines as well^36,37^, but is not used in the present work.

The processes underlying the laser printing of ZnO in the present work are mechanistically distinct from those of both, polymers and metals. Here, the laser focus of a green continuous-wave laser (rather than a femtosecond laser) is intentionally positioned below a previously laser-printed platinum wire or structure (see Fig. 1b). The plasmonic one-photon absorption of light in the platinum leads to strong local heating, which thermally drives a reaction in the ZnO ink on the wetted side of the platinum. The smooth generation of transparent ZnO in the laser printing process is shown in Supplementary Movie 1.

Compared to previously reported laser-induced hydrothermal growth of ZnO^38^, our objective is not the generation of ZnO structures by addition of additives for the control of the crystal morphology and by very long exposure times, but the controlled deposition of ZnO with precise geometrical size, high surface quality, designed morphology, and short exposure times for fast printing. Thereby laser-printed microelectronics with reproducible and controllable electrical properties are achieved.

The ZnO-“ink” reported herein is composed of Zn(NO_3_)_2_ dissolved in an ammonia solution (Supplementary Fig. 1 and “Methods”). Upon heating, first the zink(II) ammine complex dissociates. Insoluble Zn(OH)_2_ precipitates^39^ and is subsequently converted to ZnO^40^. The laser-induced local temperature field plays a critical role in this process. We have performed numerical calculations of the heat-conduction equation for these conditions, which we have calibrated by measurements on laser-printed Pt-Ag thermocouples (see Supplementary Information). We find temperature profiles (see Supplementary Figs. 2–4) that peak on the Pt wire, with peak temperatures around 100 °C, micrometer-size widths, and time constants somewhat below 100 µs.

A detailed parameter study has shown that the proper choice of the pH value of the ink is crucial to avoid dissolution of the printed material by the ink itself after the laser exposure (see Supplementary Movie 2). The pH is solely adjusted by the ammonia concentration. If the pH is too low, intermittently formed insoluble Zn(OH)_2_ would either remain or be deposited unintentionally without spatial resolution during the printing process. If the pH is too high, the ZnO structures require a higher exposure dose to form and can redissolve in the ink after exposure. For the results presented in this article (unless indicated otherwise), we use a pH value of 9.86 ± 0.01. The photothermally induced reaction locally converts the ink into polycrystalline ZnO deposited onto the platinum wire, to which it directly forms an electrical contact. After laser printing, the remaining ink is simply washed out, which forms the development step (for further details see “Methods”). We have successfully printed ZnO line structures with laser-focus speeds of up to $v=100$ µm/s The focus speeds or point exposure times, respectively, used for the making of the various devices reported below are different though and will be given for each example individually.

The overall sequence of process steps in the laser printing of heterostructures composed of Pt, ZnO, and Ag (printed in this sequence) is illustrated in Fig. 1. Note that the used lasers and illumination geometries are different in each step. For the ZnO laser printing, the shadowing by the previously laser-printed Pt lines is favorable. By contrast, laser printing of Ag does not work under shadowing conditions, such that we let the laser light impinge from the opposite side in the so-called sandwich geometry (Fig. 1c). A series of scanning-electron micrograph (SEM) images of the structures corresponding to the Pt, the ZnO, and the Ag printing step are shown in panels d–f of Fig. 1, other structures in Supplementary Figs. 5 and 6. The inset in Fig. 1e shows a magnified high-resolution electron micrograph. From 50 nanoparticles therein, we roughly estimate a mean ZnO nanoparticle size of 27 ± 5 nm. We have performed further detailed characterization of the laser-printed ZnO by scanning-electron energy-dispersive X-ray analysis (see Supplementary Figs. 7 and 8), high-resolution transmission electron microscopy, and Raman spectroscopy (refer to Supplementary Fig. 9) providing concurrent evidence that the laser-printed material is ZnO.

**Fig. 2:**
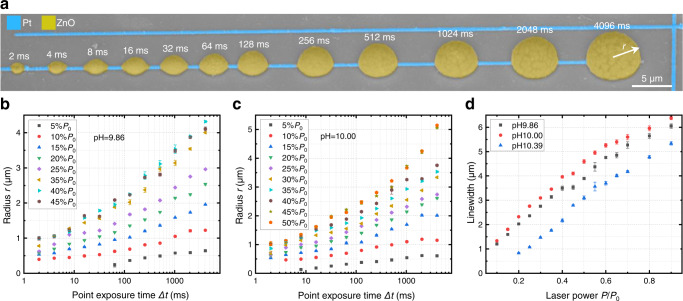
Controlling the geometry of laser-printed ZnO. **a** Scanning-electron micrograph of ZnO deposited on Pt wires by point exposure of a 532 nm wavelength continuous-wave laser. The laser power is fixed at $P=30\%\,{P}_{0}$, with ${P}_{0}=21.5\,{{{{{\rm{mW}}}}}}$, while the exposure time increases from $\Delta t=2\,{{{{{\rm{ms}}}}}}$ to $4096\,{{{{{\rm{ms}}}}}}$ from left to right. **b**, **c** Quantitative investigation of the geometrical size of laser-printed ZnO by controlling $P$ and $\Delta t$, for pH values of 9.86 ± 0.01 and 10.00 ± 0.02, respectively. **d** Linewidth of laser-printed ZnO wires versus $P$ for three different pH values of the ZnO ink, as indicated in the legend. The data points in **b**–**d** are mean values from 20 measures.

The lateral and vertical extent of the laser-printed ZnO structures can be controlled by both, the employed laser power, $P$, and the exposure time, $\Delta t$. The oblique-view electron micrograph shown in Fig. 2a reveals nearly hemispherical ZnO structures on top of a platinum wire under point exposures. Accessible radii of the ZnO hemispheres, $r$, range from *r* = 0.13 to 5.2 µm (see Fig. 2b and Supplementary Figs. 10 and 11). The radius can additionally be controlled by the pH value of the ZnO ink (compare Fig. 2b and 2c). Likewise, the width of ZnO lines on top of Pt lines can be controlled by the printing-laser power at a fixed focus velocity of, e.g., $v=20$ µm/s, and by the pH value of the ZnO ink (see Supplementary Fig. 12). For ZnO structures on top of Ag rather than Pt, the overall behavior is analogous (see Supplementary Fig. 13), yet the printing parameters need to be adjusted due to the larger heat conductivity of Ag compared to Pt.

### Laser-printed Pt–ZnO–Ag diodes

**Fig. 3:**
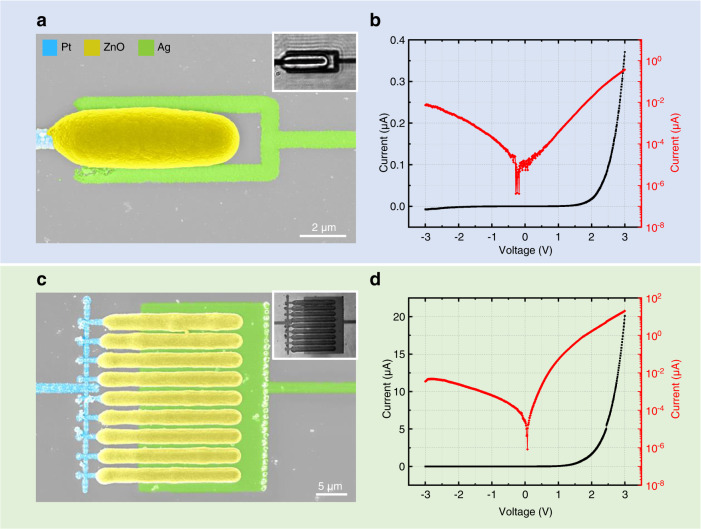
Laser-printed Pt–ZnO–Ag diodes. **a** Scanning-electron micrograph of a single diode composed of Pt and Ag wires (see coloration) and a ZnO bar in between. Inset: optical transmission micrograph of the same structure. **b** Corresponding measured current-voltage characteristics on a linear (left, black) and a logarithmic scale (right, red). **c** Scanning-electron micrograph of a diode with interdigitated metal contacts. Inset: optical transmission micrograph. **d** Current–voltage measurement for the diode in **c**.

As a first simple example of the novel capability to laser-print both, metals and semiconductors, we consider Pt–ZnO–Ag diodes in a lateral configuration. A diode composed of a platinum wire (~0.8 µm width), silver wires (~0.8 µm width), and a ZnO bar in between is shown in Fig. 3a. The channel length, i.e., the lateral spacing between the Pt and the Ag is ~0.7 µm. It is determined by the lateral width of the ZnO bar, which can be controlled by the laser-printing parameters as described in the previous section. The resulting measured current-voltage characteristics are depicted in Fig. 3b. We find a pronounced diode behavior, as expected from the different work functions of the involved materials.

In Fig. 3c, we show as another example nine parallel diodes based on interdigitated Pt and Ag lines. In analogy to the single diode in Fig. 3a, we can control the diode channel length by the ZnO thickness via the laser-printing parameters. The diode shown in Fig. 3c has an effective ratio of channel length to channel width of 176. The current-voltage curve depicted in Fig. 3d reveals currents that are about 50 times larger than for the single diode in Fig. 3b.

Yet another distinct lateral diode design is shown in Supplementary Fig. 14. In addition to these lateral diodes, we are further able to laser-print vertical diodes by virtue of the fact that the ZnO is transparent at the printing-laser wavelength. An example of such a three-layer sandwich diode structure and its current-voltage characteristics are exhibited in Supplementary Fig. 15.

### Laser-printed Pt–ZnO–Ag memristors

**Fig. 4:**
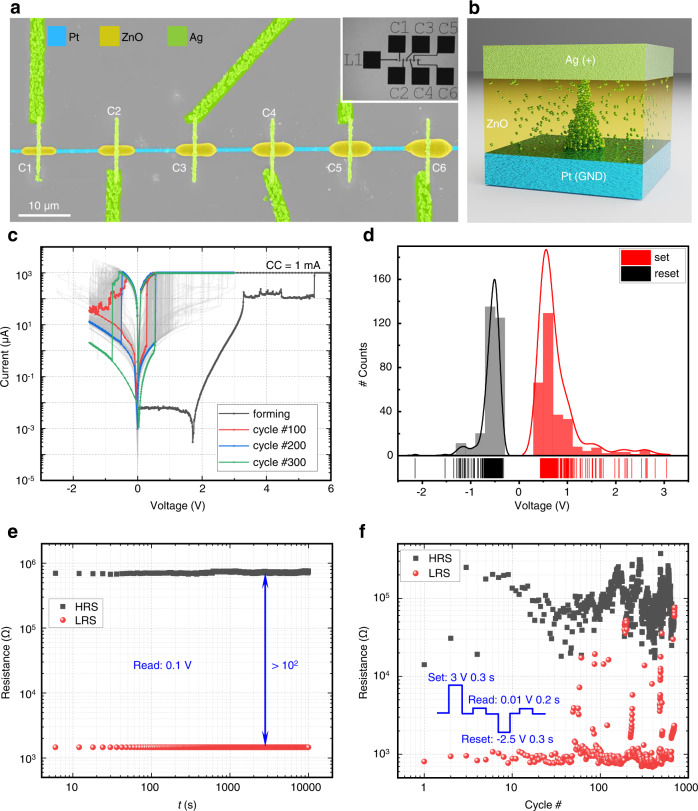
Laser-printed crossbar Pt–ZnO–Ag memristor device. **a** $1\times 6$ crossbar structure with varying ZnO linewidths, realized by controlling the laser power during laser printing. The electrical performance of the crossbar structure is determined by the dimensions of the ZnO layer. **b** Scheme of the mechanism at work. **c** Current–voltage characteristics of a single memristor for 300 cycles. The forming process (black), cycle number 100 (red), cycle number 200 (blue), and cycle number 300 (green) are emphasized by color, all other cycles are shown in gray. **d** Histograms of the set and reset voltages measured in the 300 cycles in panel **c**. **e** The stable retention performance of both resistive states on a timescale of up to ${10}^{4}{{{{{\rm{s}}}}}}$. **f**, Resistive switching endurance of a single memristor under short-pulse-voltage mode (see inset) over 700 cycles.

Throughout recent years^41–43^, memristors have emerged as candidate devices for bridging the gap between traditional volatile, fast dynamic random-access memory (RAM) and nonvolatile, yet slow electronically erasable programmable read-only memory (EEPROM). Figure 4a shows an integrated $1\times 6$ crossbar memristor array with ZnO linewidths increasing from 1.5 µm (C1) to 1.8 µm (C2), 2.2 µm (C3), 2.6 µm (C4), 3.0 µm (C5), to 3.5 µm (C6) on top of a single Pt wire with a linewidth of ~0.6 µm connected to the laser-printed contact pad L1. Six ZnO bars are printed with an equal distance of 15 μm along the Pt wire and connected to contact pads by a layer of Ag wires with a linewidth of ~0.72 µm (see whole structure in Fig. 4a inset).

The crossing of the Ag top electrode and the orthogonal Pt bottom electrode, with the ZnO sandwiched in between, leads to an effective device area that we estimate to be ~0.43 µm^2^. The material stack of this memristor is designed specifically to achieve a cation-based resistive switching^44^, in which the active Ag atoms form conducting filaments within the active layer by applying an appropriate voltage to the electrodes^45^. Through manipulation of the formation and rupture of the Ag conducting filaments in the ZnO active layer (see illustration in Fig. 4b), by applying a proper voltage to the electrodes, a typical bipolar resistive switching results for the laser-printed devices.

As an example, we electrically characterize the memristor shown in Fig. 4a. In this device, the ZnO linewidth is 1.8 µm. A positive potential is applied to the top Ag electrode, the Pt electrode is grounded. To enable the initial resistive switching in a pristine device, a forming voltage sweep ($0\,{{{{{\rm{V}}}}}}\to 8\,{{{{{\rm{V}}}}}}\to 0\,{{{{{\rm{V}}}}}}$) is applied. The black current–voltage curve in Fig. 4c shows a pronounced current increase at around 5.5 V, where it reaches the compliance current of 1 mA. This step constitutes the low-resistance state due to the formation of conducting filaments within the ZnO layer. The growth of the conducting filaments is driven by a redox reaction and the migration of Ag ions^46^.

To switch the memristor from the low-resistance state to the high-resistance state, a “reset” process is required, which is realized by a voltage sweep at the top electrode from 0 V to −1.5 V and back to 0 V. The current-voltage characteristics during the reset processes are visualized in Fig. 4c. A sudden decrease in current indicates the rupture of the conducting filaments. For the “set” operation on the same device, a voltage sweep from 0 V to +3 V and back to 0 V is applied to the top electrode. By alternatingly repeating the set and reset operations, the device can be switched between the high-resistance and the low-resistance state over 300 cycles. The 300 current-voltage curves of the set and reset processes are shown in Fig. 4c on a semi-logarithmic scale, from which an on/off ratio of about ${10}^{2}$ can be seen. A histogram of the set and reset voltages, respectively extracted from the current-voltage curves in Fig. 4c, is depicted in Fig. 4d. We find well-separated maxima at $0.77\,{{{{{\rm{V}}}}}}\pm 0.45\,{{{{{\rm{V}}}}}}$ for the set operation and at $-0.58\,{{{{{\rm{V}}}}}}\pm 0.20\,{{{{{\rm{V}}}}}}$ for the reset operation, respectively.

A high retention time is a crucial factor for reliable memristor-based circuits and systems^47–49^. To characterize the retention performance, the memristor is switched to the high-resistance and the low-resistance state, respectively, and the resistance of the device is measured every 6 s by applying a voltage pulse with a width of 0.2 s and a height of 0.1 V to the top electrode over a total timespan of 10^4^ s. As can be seen from Fig. 4e, both states are stable over the entire measurement period, with no sign of degradation.

To examine the dynamic performance of the device when subject to voltage pulses for programming, we use the waveform shown in the inset of Fig. 4f. This waveform consists of a set voltage pulse and a reset voltage pulse with the indicated widths and heights. After each voltage pulse operation, the resistance state of the device is read out by a small voltage pulse (width 0.2 s, height 0.01 V) while it is repeatedly switched between the high-resistance and the low-resistance state over 700 times (see Fig. 4f). Only a few set operations have failed during the 700 switching cycles. This behavior of the fully laser-printed memristor can be compared with other approaches. Compared to solution-processed devices, the endurance in terms of number of switching cycles of our device is better than that of most devices from other reports^41^.

We note that the size of the semiconducting ZnO layer of a memristor in a crossbar can be flexibly controlled by the laser printing parameters (see above), thereby allowing to tailor the electrical behavior, as shown in Supplementary Fig. 16. Using this flexibility, we can integrate multiple elements with dissimilar geometrical and hence dissimilar electrical performance (Supplementary Fig. 17). Beyond single crosspoint structures, the three-layer memristor can be patterned to form large-area structures or other complex patterns. As an example, a sandwich-mode memristor with a footprint of 30 × 30 µm^2^ is demonstrated and characterized in Supplementary Fig. 18.

### Laser-printed Pt–ZnO–Ag security circuits

**Fig. 5:**
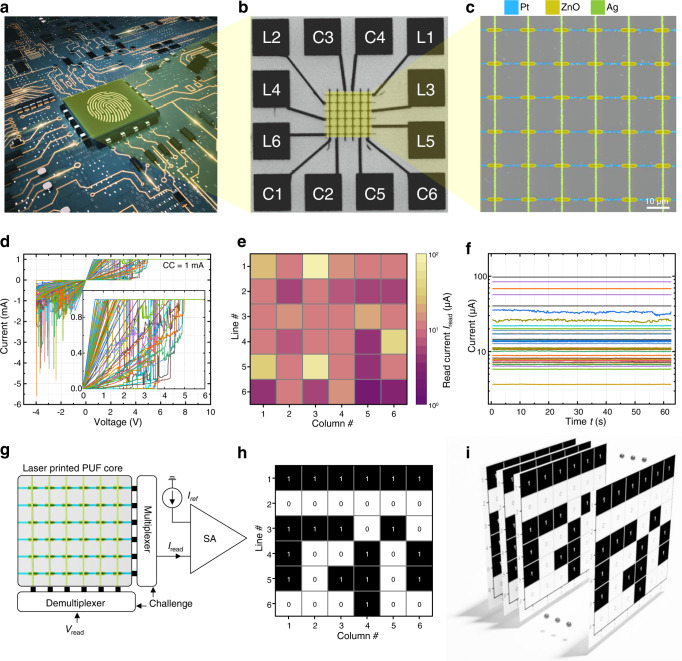
Laser-printed Pt-ZnO-Ag security circuits. **a** Schematic of an embedded hardware-based security primitive, based on a physically unclonable function (PUF). **b** Optical micrograph of a laser-printed $6\times 6$ crossbar array with contact pads. **c** Scanning-electron micrograph of a laser-printed $6\times 6$ crossbar array, showing a period of 1.5 µm in both, horizontal and vertical directions. The memristive devices are located at the wire crossing junctions. **d** Activation of the 36 memristor cells by sweeping a voltage from $0\,{{{{{\rm{V}}}}}}\to 10\,{{{{{\rm{V}}}}}}\to -4\,{{{{{\rm{V}}}}}}\to 0\,{{{{{\rm{V}}}}}}$. The inset shows the set process of the 36 memristor cells for positive voltages, with the compliance current set to $1{{{{{\rm{mA}}}}}}$. **e** False-color representation of the read-out currents ${I}_{{{{{{\rm{read}}}}}}}$ from the $6\times 6$ array. **f** Retention performance of ${I}_{{{{{{\rm{read}}}}}}}$ over a timespan of 60 s. **g** Circuit architecture around the core of the memristor-PUF. **h** Bit-array distribution corresponding to the $6\times 6$ crossbar array. **i** Schematic of the evaluated bit errors over 300 cycles.

Next, we exploit the stochastic nature of the laser-printed memristors in a $6\times 6$ crossbar architecture shown in Fig. 5a–c. The circuit is utilized as physically unclonable function, which is a hardware-based security primitive. This function generates a unique response upon stimulation by a challenge, comparable to a human fingerprint^50^. Herein, the variability of the memristors’ behavior due to stochastic formation and rupture of conducting filament paths is key^45,47,51^. For the initialization of the laser-printed memristor-based crossbar physically unclonable function, each device in the array is initially “formed” and then “reset”, using a floating bias scheme. The initial forming and reset processes of the 36 memristor cells are shown in Fig. 5d and Supplementary Fig. 19. After the initialization, the crossbar forms an interconnected resistive network, with varying resistances of the corresponding memristor cells and the laser-printed interconnects.

After the initialization of the crossbar, each memristor cell is addressed by applying a read-out voltage of $0.1\,{{{{{\rm{V}}}}}}$ and measuring the corresponding read-out current ${I}_{{{{{{\rm{read}}}}}}}$ in a floating bias scheme. The read-out current not only depends on the conductance of the selected cell, but also on the sum of all sneak-path currents^52^ of unselected devices within the full crossbar structure. To investigate noise effects on response stability, we have performed read-out in 200 ms time intervals over 300 cycles for each device. Figure 5e shows the obtained ${I}_{{{{{{\rm{read}}}}}}}$ values in false-color representation, and Fig. 5f the temporal stability of the selected cells. The cumulative distribution function of the ${I}_{{{{{{\rm{read}}}}}}}$ values is shown in Supplementary Fig. 20. For the complete architecture around the core of the physically unclonable function, a control logic as illustrated in Fig. 5g is required. Here, we emulate this logic (see “Methods”) by a validated setup, which was previously used in another work^53^. The extracted bit patterns illustrated in Fig. 5h demonstrate that the laser-printed $6\times 6$ array of Pt–ZnO–Ag memristors shown in Fig. 5b, c represents a fully functional security circuit based on a physically unclonable function. Furthermore, no bit errors (BE = 0%) of the PUF response occur over 300 iterations for the investigated crossbar. The bit error calculation is schematically shown in Fig. 5i (also see “Methods”).

Finally, we emphasize that laser printing can not only be used as a stand-alone technology, but can also readily be combined with other printing modalities such as, e.g., inkjet printing, thereby significantly expanding the toolbox of additive-manufacturing possibilities for electronics. As a proof-of-principle demonstration, Supplementary Figs. 21 and 22 show results for fully functional transistors entirely made by hybrid laser and inkjet printing.

## Conclusions

We provide a number of proof-of-principle demonstrations for direct laser printing of functional electronic devices and entire electronic circuits with minimum feature sizes down to <1 µm. A crucial prerequisite for this breakthrough has been the development of an inorganic semiconductor (ZnO) ink for laser printing via photothermal synthesis. This ink and the associated printing modalities are conceptually distinct from both laser printing of polymers and metals. The advantages of direct laser printing of memristors and circuits based thereupon are the complete additive manufacturability, the possibility of 2D and 3D integration in one process, and the flexibility to design each individual device according to the intended electrical behavior. For example, it should be possible to realize different bottom and top metal electrodes, various semiconductors and varying dimensions in one design. Also flexible substrates are feasible. This especially implies that no high-vacuum deposition and no standard cleanroom lithographic processes are needed for any of the devices presented in this work.

## Methods

### Materials

#### Pt ink

A 500 mM stock solution of ammonium iron oxalate trihydrate ((NH_4_)_3_[Fe(C_2_O_4_)_3_]·3H_2_O, Alfa Aesar, 98%), and a 70 mM stock solution of ammonium tetrachloroplatinate ((NH_4_)_2_[PtCl_4_], Alfa Aesar, 99.9% metal basis) in deionized water (conductivity 16–18 MΩ) were prepared and stored in the dark at 4 °C. The platinum ink (a 1:1 mixture by volume of (NH_4_)_3_[Fe(C_2_O_4_)_3_] and (NH_4_)_2_[PtCl_4_]) stock solutions was always freshly prepared prior to the laser printing experiments.

#### Ag ink

An aqueous solution of 83 mM silver nitrate (AgNO_3_, Alfa Aesar, 99.9+% metal basis) and 62 mM trisodium citrate (Carl Roth, 99% p.a. ACS) was prepared. ~14 M aqueous ammonia solution (28–30 %, Merck, ACS Reag. Ph. Eur.) was added dropwise under stirring until the intermittently formed precipitate redissolved and a homogeneous solution was obtained.

#### ZnO ink

To obtain a ZnO ink with both a precise pH and Zn^2+^ concentration, the addition of ammonia must be conducted while monitoring the pH and the temperature. The pH values were determined using a Mettler-Toledo Seven Easy pH meter equipped with a Mettler-Toledo InLAB Science Pro-ISM electrode (inside/outside electrolyte 3.00 mol L^−1^ KCl) equipped with a built-in temperature sensor (NTC). The built-in temperature compensation was used to arrive at the errors quoted in the main text. The electrode was calibrated by three-point calibration using pH 4.00, 7.00 and 10.00 calibrants (Roti®Calipure, Carl Roth).

Zink nitrate hexahydrate (Zn(NO_3_)_2_·6 H_2_O, Alfa Aesar, >98%) was dissolved in deionized water (conductivity 16–18 MΩ, pH = 6.30) at ambient temperature. ~14 M aqueous ammonia (28-30 %, Merck, ACS Reag. Ph. Eur.) was added dropwise until the intermittently formed white precipitate was almost completely redissolved. Subsequently, the solution was stirred at 20 °C for 15 min. Additional ammonia was added dropwise until the desired pH value was reached. Afterwards, the solution was filled into a volumetric flask and deionized water was added. The pH was measured again and eventually adjusted by adding a few drops of ammonia. Thereby the concentration of Zn^2+^ was fixed to 0.4 M and the pH value was adjusted to 9.86 ± 0.01, 10.00 ± 0.02 and 10.39 ± 0.02, respectively. Finally, the ink was passed through a Satorius Minisart syringe filter (hydrophilic, 0.2 μm).

### Sample preparation

Cover glasses were silanized for better adhesion of the platinum particles to the amino-functionalized substrate surface during the laser-printing process. Following an oxygen-plasma treatment for 10 min, the silanization was performed by immersing clean cover glasses in vials containing a solution of (3-aminopropyl) triethoxysilane dissolved in toluene (0.2% vol) for 60 min under ambient conditions. A polydimethylsiloxane (PDMS) frame with a height of about 1 mm was adhered to the silanized glass substrate, forming a reservoir. A droplet of ink (Pt, ZnO, or Ag) was placed in the middle of the PDMS reservoir and then sealed by covering a second PDMS piece to prevent any evaporation of solvent during the laser-printing process.

### Instrumentation

A Ti:Sa femtosecond laser (Coherent, Chameleon Ultra II) was used for multi-photon printing of Pt and Ag. A 532 nm continuous-wave laser (Coherent, Verdi-V5) was used for photothermal synthesis of ZnO. The used power of the two lasers was controlled with two acousto-optic modulators (AA Opto Electronic, MTS40-A3–750.850 and MT80-A1,5-VIS, respectively). The two laser beams were combined by means of a dichroic mirror and focused onto the ink-substrate interface by using an oil-immersion microscope objective lens (Zeiss, Plan-APOCHROMAT 100×/1.4 Oil) for Pt, and a water-immersion microscope objective lens (Zeiss, LD C-APOCHROMAT 100×/1.25 W) for ZnO and Ag. The objectives were mounted on a piezoelectric stage (Physik Instrumente, P-733.ZCL) with 100 μm travel to translate the focus along the optical axis. The sample was translated horizontally using a combination of a piezo stage (Physik Instrumente, P-734.2CL, 100 μm × 100 μm travel) and a motorized stage (Physik Instrumente, P-M-686, 25 mm × 25 mm travel). It is important to find the correct position of the ink-substrate interface during the writing procedure, which was realized with high accuracy via a confocal detection scheme. In this interface-finding process, we scanned a focused probe laser (at 675 nm wavelength) with respect to the glass substrate along the $z$-direction in a range of a few micrometers. The position of the maximum of the reflected light power versus $z$-position was taken as the interface position. To monitor the printing process in situ, we used a camera and light illumination in transmission by a red light-emitting diode.

### Laser printing of microelectronic devices

All diodes, memristors, and security circuits presented here were printed in the sequence Pt, ZnO, and Ag. Uniform and smooth Pt structures in the first step facilitate the subsequent ZnO printing. Otherwise, sharp bumps or spikes on the Pt wires may lead to heat accumulation and bubble generation, hindering the smooth and reproducible printing of ZnO. To print uniform Pt wires, we used laser powers in front of the microscope objective lens entrance pupil of 0.5–1.2 mW and focus speeds between 5 and 10 μm/s. After laser exposure, the samples were washed for 5 min in pure water. Thereafter, they were blown dry using nitrogen gas. Next, the ZnO ink was drop-cast onto the Pt structures. The laser powers (532 nm wavelength, continuous-wave) used for the ZnO printing process refer to the entrance pupil of the microscope objective lens and are quoted in percentages of the reference power ${P}_{0}=21.5\,{{{{{\rm{mW}}}}}}$. The exposure times for the point exposure exhibited in Fig. 2 vary from 2 ms to 4096 ms, as indicated there. For the printing of ZnO lines or patterns, we used a fixed focus velocity of $v=20$ µm/s. The samples were first washed in water for 5 min, and then in ethanol for 5 min. The samples were dried by a flow of nitrogen gas before they were immersed in the Ag ink. For the laser-printing of Ag wires, the laser powers were in the range of 0.35–0.7 mW and the focus speeds were in the range $v=10{-}20$ µm/s. The large Ag electrode pads with footprints 70 μm × 70 μm shown in Figs. 4 and 5 were printed at a higher focus velocity of $v=50$ µm/s.

After completing the fabrication in an ordinary laboratory (no cleanroom environment), the devices were stored under ambient conditions in the laboratory. This included exposing them to large humidity in the summer time. In some cases, samples were stored for weeks before performing the characterization measurements. If necessary, the devices could be protected by standard encapsulating (e.g., parylene or expoxy) or even by a laser-printed polymer.

### Material characterization

Samples were characterized by a Zeiss Leo 1530 scanning-electron microscope operating at 10.0 keV electron energy. Prior to inspection, the samples were coated by an 8 nm thin gold layer. A double-corrected ThermoFisher Themis-Z equipped with a Super-X EDX detector was used to characterize microstructure and obtain chemical information including the selected-area electron diffraction (SAED) and scanning-transmission-electron microscopy energy-dispersive X-ray spectroscopy (STEM-EDX). The microscope was operated at an accelerating voltage of 300 kV. The TEM measurements were performed by locating the ZnO sample on a lacey-carbon-coated copper grid. The TEM grids with samples loading were loaded onto a FEI double tilt holder. The resolution of STEM-EDX used in this manuscript was 6.548 nm. A Renishaw inVia confocal Raman Microscope was used to record Raman spectra. We used a magnification of 100×. The samples were irradiated with a continuous-wave laser at 532 nm wavelength. Data shown in the Supplementary Information are the average over 10 individual measurements with 30 s acquisition time each.

### Electronic characterization

Room-temperature current-voltage characteristics of the diodes, memristors, and transistors were recorded by a probe station (Cascade, MPS150) combined with a semiconductor parameter analyzer (Keithley, 4200A SCS).

### PUF core initialization

To enable device forming, a voltage sweep from $0\,{{{{{\rm{V}}}}}}\to 10\,{{{{{\rm{V}}}}}}\to 0\,{{{{{\rm{V}}}}}}$ with a 10 mV step size was applied to the Ag-based top electrode, with the corresponding Pt-based bottom electrode grounded. The compliance current for the forming process was set to 1 mA. To reset the device, a negative voltage sweep from $0\,{{{{{\rm{V}}}}}}\to -4\,{{{{{\rm{V}}}}}}\to 0\,{{{{{\rm{V}}}}}}$, also with a 10 mV step size, was applied to the devices. For forming, reset, and read operations, a floating bias scheme was used, where all unselected lines and columns were left floating.

### PUF control logic

The control logic of the PUF contained a multiplexer (MUX) and demultiplexer (DEMUX) for signal routing. By applying the challenge, which holds the DEMUX and MUX addresses for line and column selection, each memristor cell was selected, by applying *V*_read_ and routing *I*_read_ to the sensing amplifier input terminal. The iteration started at the uppermost line and went over all columns and then to the next line. This was continued, until all cells were addressed and the full challenge response pair (CRP) was built, which led to a 36-bit wide response.

The selected cell’s *I*_read_ was compared to a reference current, *I*_ref_, by a sense amplifier (SA), as shown in Fig. 5g. Based on which current exceeds the other, the sense amplifier generated an output (*r*_*k*_) logic “0” or logic “1”, according to Eq. (1):

1 $${r}_{k}=\{1\,{{{{{\rm{if}}}}}}\,{I}_{{{{{{\rm{read}}}}}}} \, > \,{I}_{{{{{{\rm{ref}}}}}}},\, 0\,{{{{{\rm{if}}}}}}\,{I}_{{{{{{\rm{read}}}}}}} \, < \,{I}_{{{{{{\rm{ref}}}}}}}\}.$$

With this method, a *L*_max_ = *M* long PUF response was generated, where *L*_max_ was the maximum obtainable response length of the PUF, and *M* the amount of incorporated memristor cells.

As a suitable value for the reference current *I*_ref_, the median of the experimentally obtained *I*_read_ values was used, which was 13.21 µA for the laser-printed crossbar structure shown in Fig. 5. For the sense amplifier, the minimal current difference which can be sensed was assumed to be 100 nA. Lower differences between currents were not distinguished but could potentially lead to a bias in the PUF response. The obtained bit-distribution across the array and the obtained 36-bit wide response *R* = (*r*_1_, *r*_2_, *r*_3_, …, *r*_*k*_) = (111111000000111010100101101101000100) are shown in Fig. 5h.

### Bit errors

With the obtained read currents over time, the PUF core’s bit errors were calculated, according to Eq. (2), where *R*_ref,*n*_ being the *n*-th (here, *n* = 1), *L*-bit long reference response and *R*′_*n,w*_, the *W*-times (*W* = 300) regenerated response^53^.

2 $${{{{{{\rm{BE}}}}}}}_{n}=\frac{1}{W}\mathop{\sum }\limits_{w=1}^{W}\frac{{{{{{\rm{HD}}}}}}({R}_{{{{{{\rm{ref}}}}}},n},\,{{R}^\prime}_{n,w})}{L}\cdot 100\%$$

## Supplementary information

Supplementary Information

Peer Review File

Description of Additional Supplementary Files

Supplementary Movie 1

Supplementary Movie 2

## Data Availability

The data underlying the figures within this paper, COMSOL files for thermal simulation, and Supplementary Information are published on the open-access data repository of the Karlsruhe Institute of Technology (10.35097/794).
