# Supplementary material for: Laser printed microelectronics
Source: Nat Commun. 2023 Feb 27;14:1103. doi: 10.1038/s41467-023-36722-7 (PMC9968718; doi:10.1038/s41467-023-36722-7)
Supplement: Supplementary file 1 — Supplementary Information [file 41467_2023_36722_MOESM1_ESM.pdf]

Supplementary Information

Laser Printed Microelectronics

Liang Yang[1,2,#,*], Hongrong Hu[1], Alexander Scholz[1], Florian Feist[1], Gabriel Cadilha Marques[1], Steven Kraus[1,2], Niklas Maximilian Bojanowski[1], Eva Blasco[1,3,4], Christopher Barner-Kowollik[1,5,6], Jasmin Aghassi-Hagmann[1], Martin Wegener[1,2, *]

[1]Institute of Nanotechnology (INT), Karlsruhe Institute of Technology (KIT), 76128 Karlsruhe, Germany

[2]Institute of Applied Physics (APH), Karlsruhe Institute of Technology (KIT), 76128 Karlsruhe, Germany

[3]Institut für Organische Chemie, Ruprecht-Karls-Universität Heidelberg, Im Neuenheimer Feld 270, 69120 Heidelberg, Germany

[4]Centre for Advanced Materials (CAM), Ruprecht-Karls-Universität Heidelberg, Im Neuenheimer Feld 225 and 270, 69120 Heidelberg, Germany

[5]School of Chemistry and Physics, Queensland University of Technology (QUT), 2 George Street, Brisbane, QLD 4000, Australia

[6]Centre for Materials Science, Queensland University of Technology (QUT), 2 George Street, Brisbane, QLD 4000, Australia

[#]Present address: Suzhou Institute for Advanced Research, University of Science and Technology of China (USTC), 215127 Suzhou, China

E-mail: martin.wegener@kit.edu; ygliang@ustc.edu.cn

Contents

Supplementary **Figure S1**: Photographs of flasks with Pt ink, ZnO ink, and Ag ink 4

Supplementary **Figure S2**: Measured temperature in laser printing process by laser printed Pt-Ag thermocouple 5

Supplementary **Figure S3**: Simulated temperature field generated in laser printing process ……... 7

Supplementary **Figure S4**: Simulated temperature profile along and perpendicular to metal line ……... 9

Supplementary **Figure S5**: Direct laser printing of complex multi-material patterns............... 10

Supplementary **Figure S6**: Optical images of ZnO patterns 11

Supplementary **Figure S7**: EDX map of laser printed ZnO on Pt wire 12

Supplementary **Figure S8**: EDX spectrum of ZnO fragments... 13

Supplementary **Figure S9**: High resolution TEM and Raman spectrum of printed ZnO 14

Supplementary **Figure S10**: Oblique-view SEM of ZnO hemispheres from point exposures ...16

Supplementary **Figure S11**: SEM of ZnO structures for different exposure doses …............. 17

Supplementary **Figure S12**: SEM of ZnO lines for with different laser power...….................. 18

Supplementary **Figure S13**: ZnO printing on Ag wires.. 19

Supplementary **Figure S14**: Gallery of multi-material structures.. 20

Supplementary **Figure S15**: Vertical diode composed by Pt, ZnO, and Ag ………………..... 22

Supplementary **Figure S16**: Modulation of electronic performance of 1×6 crossbar structures by controlling the ZnO layer thickness 23

Supplementary **Figure S17**: Tunable electronic performance of 3×3 crossbar structures by controlling the ZnO layer thickness.. 25

Supplementary **Figure S18**: Sandwich-mode memristor.. 27

Supplementary **Figure S19**: I-V Characterization of all the 6×6 crossbar memristors in an array.. 30

Supplementary **Figure S20**: Empirical cumulative distribution of I_{read} of 6×6 crossbar structure.. 31

Supplementary **Figure S21**: Electronic performance of Pt-ZnO-Pt structure before further inkjet printing.. 32

Supplementary **Figure S22**: Transistor from the combination of laser- and inkjet printing ... 34

Supplementary **Figure S23**: Not-colored versions of all electron micrographs of main paper..36

References..37

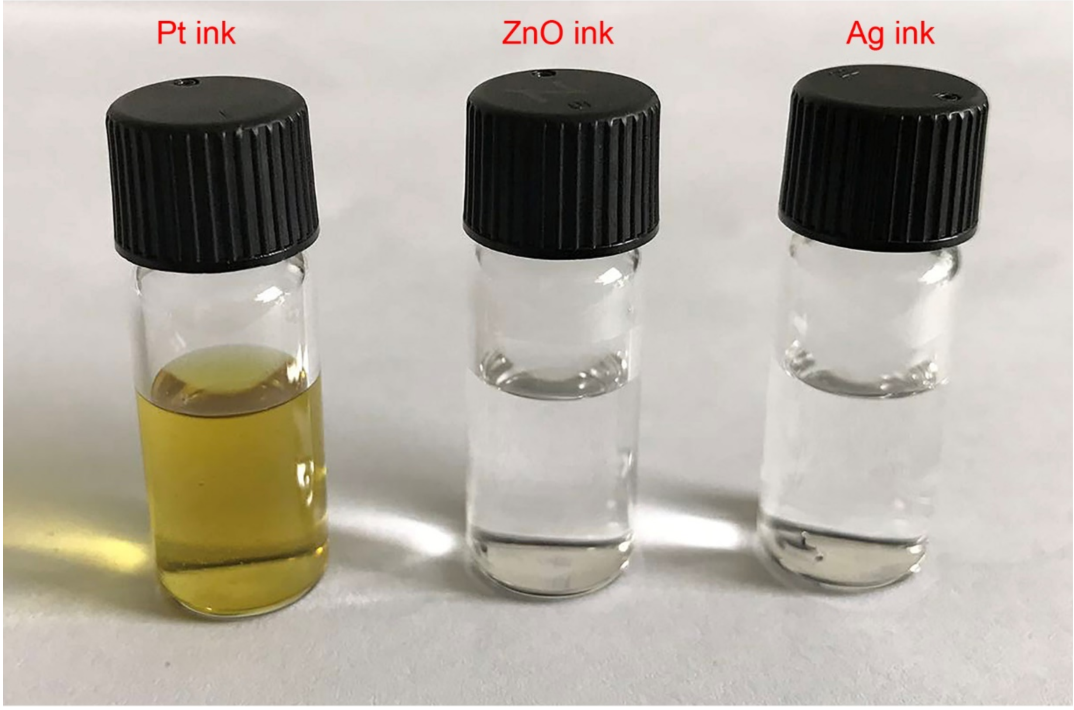

Figure S1: Photographs of flasks containing the Pt ink, ZnO ink, and Ag ink. All inks are water based and transparent. The Pt ink (left) exhibits a brownish color, while the ZnO (middle) and Ag (right) inks are colorless.

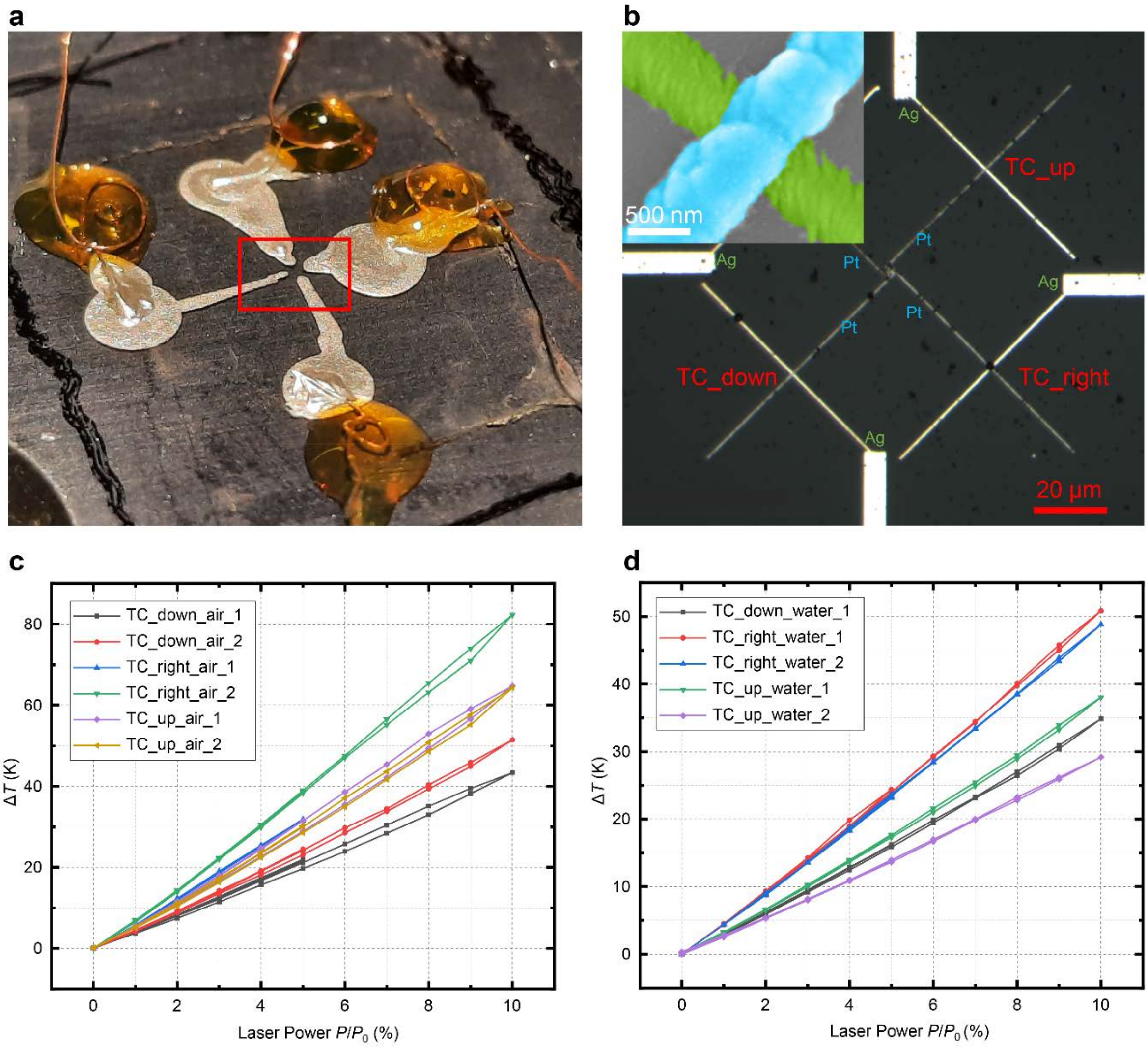

Figure S2: Temperature measured by laser-printed Pt-Ag thermocouples. a, Photograph of a thermocouple sample. **b**, Optical image of 4 thermocouples with laser-printed Pt-Ag junctions. The inset shows a magnified view onto a Pt-Ag junction. **c**, Measured temperature increase under laser exposure in air environment at room temperature. The couples are exposed to a laser until steady-state is achieved, at which point the voltage is measured. The reference laser power is $P_0 = 21.5$ mW. **d**, Measured temperature increase under laser exposure in water environment at room temperature.

Pt-Ag thermocouples have been fabricated by laser-printing a silver line perpendicular to a platinum line. Upon positioning a laser focus onto the thermocouple, a thermo-voltage results,

which is then amplified and detected by a high-impedance voltmeter. Due to the junction's finite size, the temperature varies within. Hence, the measured voltage merely provides a rough estimate of the temperatures at work. Combined with an ambient temperature of 22°C, the measurements performed in air convert to absolute temperatures between 60°C and 100°C, resulting in temperatures between 50°C and 110°C throughout the couple's junctions at a laser power of 10%P_0. For converting the measured thermo-voltages into temperature, we have used the literature value for the Seebeck coefficient of silver relative to platinum of 6.5 µV/K. As usual for macroscopic thermocouples, independent calibration experiments have not been performed. The multiple experiments performed on a single thermocouple shown in Fig. S2 reveal significant variations. To obtain reliable results, the position of the laser focus is chosen by maximizing the thermocouple signal. However, due to the size of the laser focus, which is on the order of the metal line width, small misplacements result in significant variations of the result. Additionally, the electrical and thermal conductivities of the laser-printed platinum and silver lines change under laser irradiation due to an "annealing process" induced by the laser focus.

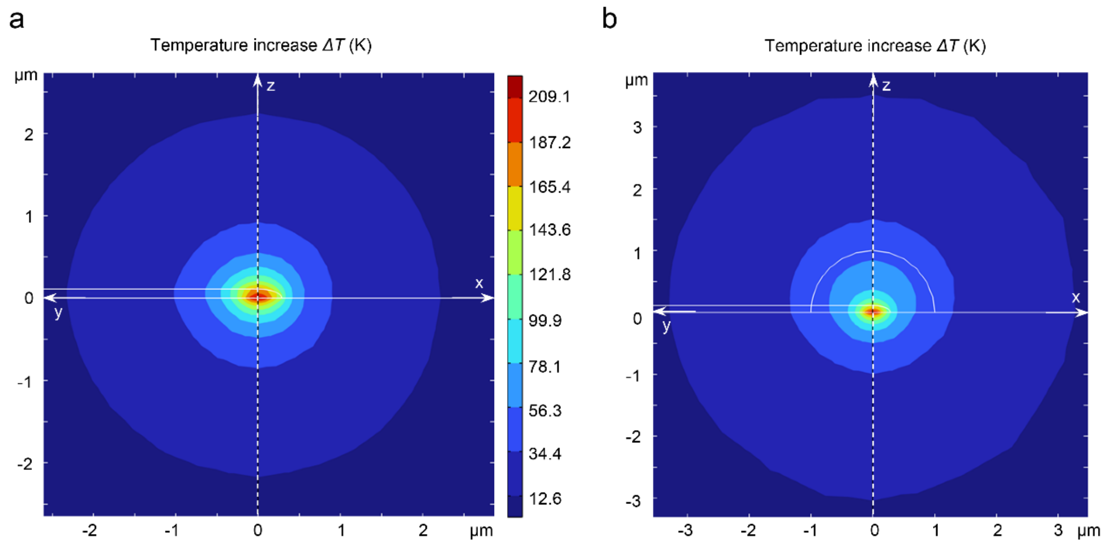

Figure S3: COMSOL simulations of temperature fields generated in the laser-printing process. a corresponds to a bare platinum line, while **b** corresponds to a platinum line with a hemisphere of ZnO on top. Heat maps after 25 ms of exposure are shown, taken at $P/P_0 = 20\%$ and with a ZnO thickness of 1 μm in **b**. The heat maps within the *yz*-plane (parallel to metal line) and in the *xz*-plane (perpendicular to metal line) are shown one the left and right of each panel, respectively. The COMSOL files used for these temperature-field simulations as well as for those shown in Fig. S4 are provided via a link (see data availability section).

As a first step, a simulation of the thermocouple corresponding to the "TC_right_air_1" experiment of Fig. S2c is performed. With its structural parameters, as well as data on the glass substrates' and as water-approximated ink's thermal properties, the simulations are calibrated to find the metal lines' thermal conductivities. Since they are composed of nanoparticles, their thermal conductivity is lower than that of the bulk material. Adjusting these to fit the experimental data resulted in thermal conductivities of 1/18.3 of those of the bulk materials. In these experiments, no ZnO is present. As the presence of the ZnO changes the temperature profiles, an estimate for the ZnO thermal conductivity is needed. We use the same fraction of the literature bulk ZnO value.

Table S1: parameters used for temperature field simulations.

Parameters	Thermal conductivity	Heat capacity	Density
**Platinum**	3.88[W/(m*K)]	133[J/(kg*K)]	21450[kg/m^3]
**Glass (Borosilicate glass, D263)**	0.96[W/(m*K)]	820[J/(kg*K)]	2510[kg/m^3]
**Water**	0.6[W/(m*K)]	4184.4[J/(kg*K)]	998[kg/m^3]
**ZnO (T<412)**	(255.4887-1.665393*T^1+0.006492632*T^2-1.361659E-5*T^3+1.102392E-8*T^4)/18.3 [W/(m*K)]	(41.5787678+3.00914995*T^1-0.00680346851*T^2+7.3427108E-6*T^3-3.02934738E-9*T^4)/18.3 [J/(kg*K)]	(5672.032-0.001431334*T^1-1.451409E-4*T^2+7.596009E-8*T^3-1.590979E-11*T^4)/18.3 [kg/m^3]
**ZnO (T>412)**	(277.1409-1.140114*T^1+0.001840955*T^2-1.338889E-6*T^3+3.666218E-10*T^4)/18.3 [W/(m*K)]	(41.5787678+3.00914995*T^1-0.00680346851*T^2+7.3427108E-6*T^3-3.02934738E-9*T^4)/18.3 [J/(kg*K)]	(5672.032-0.001431334*T^1-1.451409E-4*T^2+7.596009E-8*T^3-1.590979E-11*T^4)/18.3 [kg/m^3]
**Ambient temperature**	295.15 K		

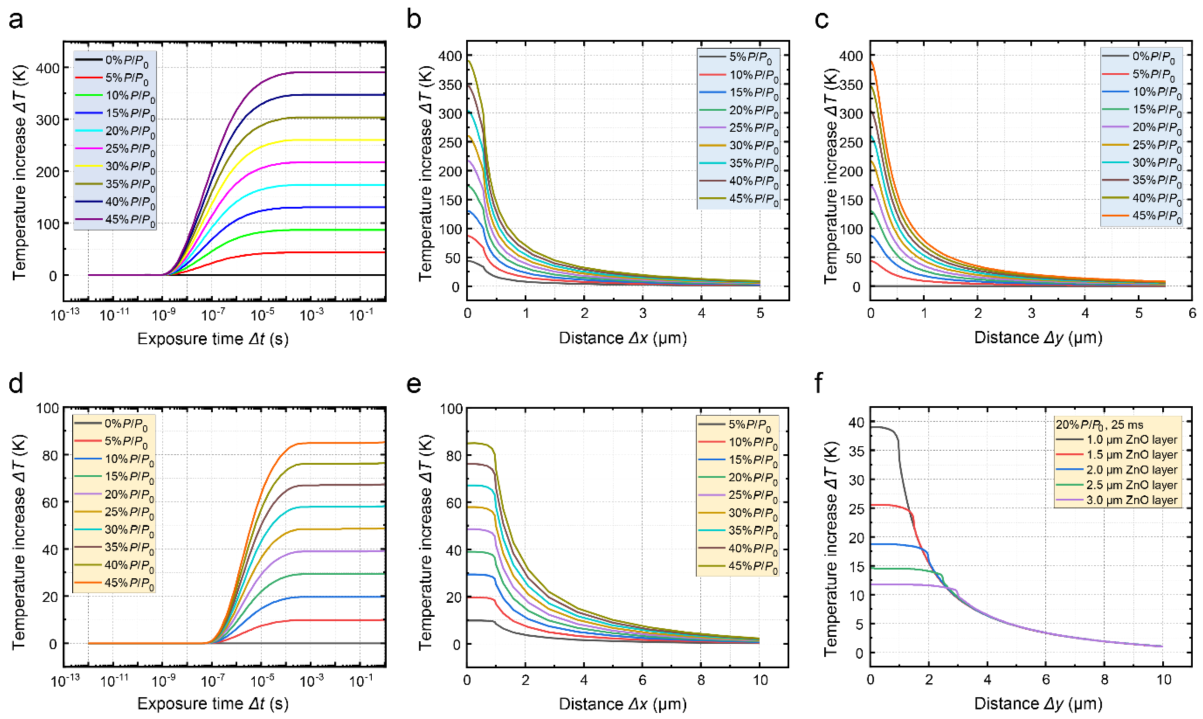

Figure S4: Temperature profile along metal-ink and ZnO-ink interface. a, **b**, and **c** correspond to a bare platinum line, while **d**, **e**, and **f** correspond to a platinum line with a hemisphere of ZnO with a thickness of 1 μm on top. **a**, and **d** show the temperature developments over time for various laser powers. The quoted temperatures refer to the hottest point exposed to the ink, which lies directly above the laser at the metal/ink or ZnO/ink interface. The temperatures reach 95% of the maximum after 10 μs and 70 μs in **a** and **d**, respectively. **b** and **e** show temperature profiles perpendicular to the printed metal line collected after 25 ms exposure time. **c** and **f** show temperature profiles along a printed metal line after 25 ms exposure time. The temperatures refer to the metal/ink or ZnO/ink interface, respectively. In **f**, instead of power sweeping, the thickness of ZnO layer increases from 1 μm to 3 μm, for P/P_0 = 20% and 25 ms exposure time. From the linearity of the heat—conduction equation it is clear that the temperature profiles are proportional to the laser power. They decay roughly exponentially in the radial direction. This exponential decay shifts in space as the ZnO thickness increases.

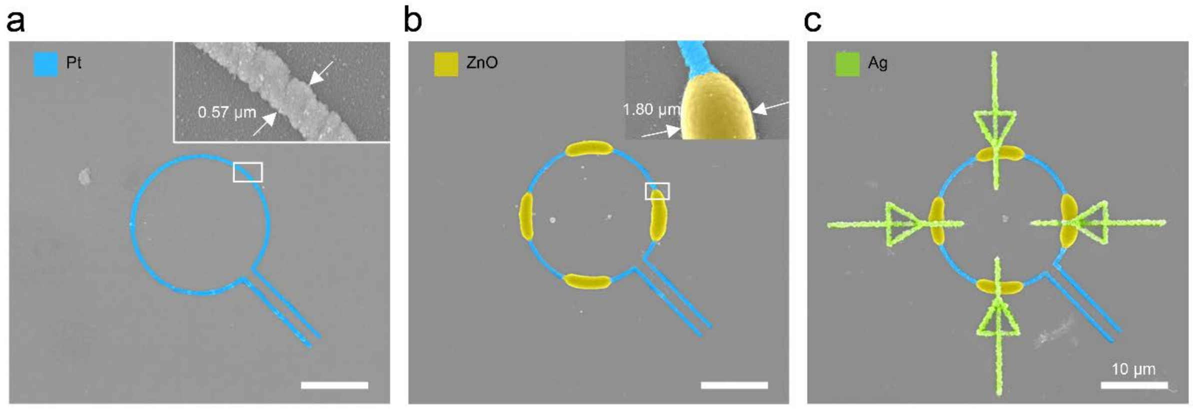

Figure S5: Direct laser-printing of complex multi-material patterns. **a**, SEM image of a circular Pt pattern printed by multi-photon metal reduction. The inset shows a magnified view of a Pt wire with 0.57 µm linewidth. **b**, ZnO can be deposited at arbitrary positions by selectively focusing the laser beam on the existing Pt pattern. The inset shows the edge between Pt and ZnO. Here, the linewidth is 1.80 µm. The linewidth can be controlled by the laser-printing parameters. **c**, A Ag pattern is printed. Note that the Ag line starts far away from the ZnO and extends over the ZnO towards the interior of the Pt ring. The figure serves as another example demonstrating that nearly arbitrary patterns composed of Pt, Ag, and ZnO can be laser-printed in a controlled way.

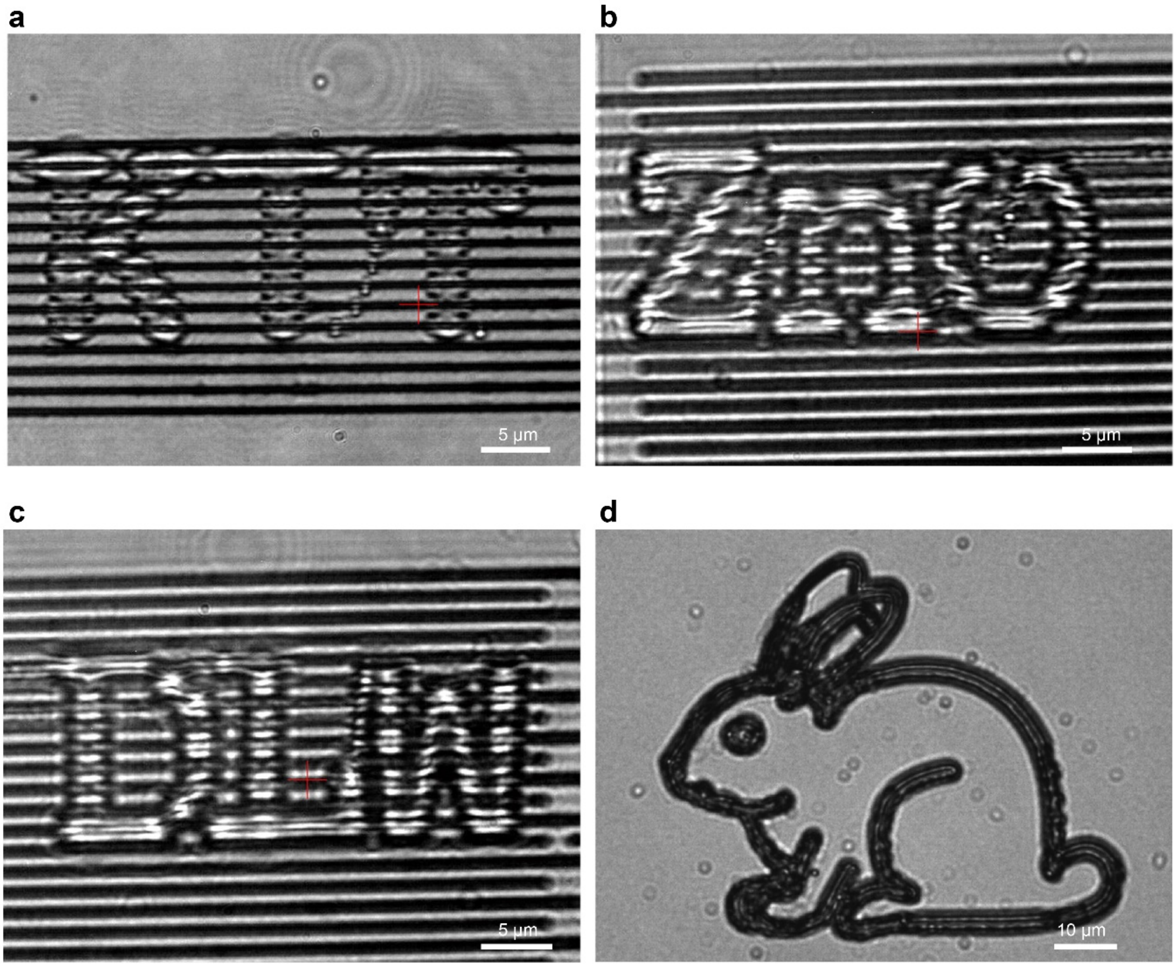

Figure S6: Optical images of ZnO patterns. In **a**, **b**, and **c**, “KIT”, “ZnO”, and “DLW” ZnO patterns are laser-printed on parallel Pt wires with a pitch of 1.5 µm. The Pt wires underneath the ZnO are clearly visible, indicating that the ZnO layer is transparent in the visible spectral regime. In **d**, Pt wires forming a rabbit pattern have been laser-printed in a first step. In the next step, a ZnO structure is printed on top. This structure covers the entire Pt line.

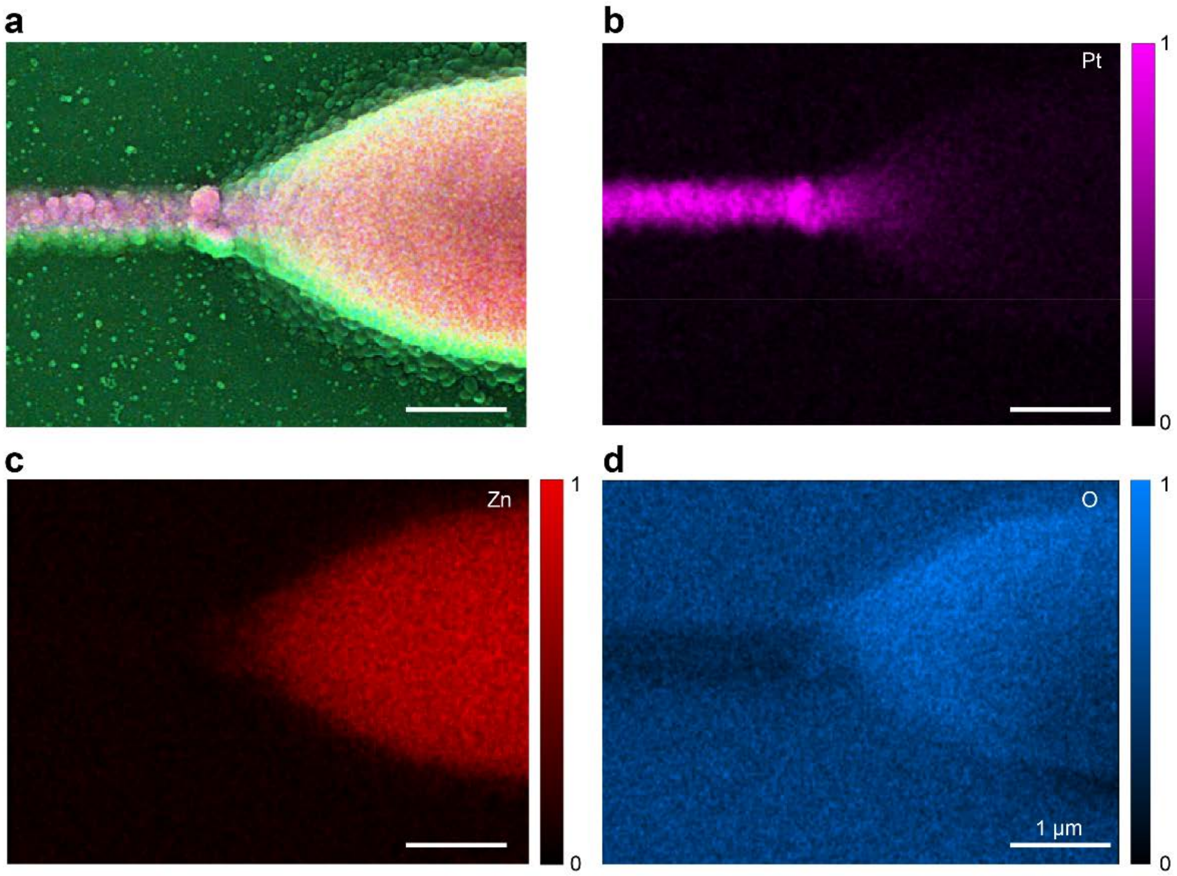

Figure S7: EDX map of laser-printed ZnO on top of a Pt wire. a, Scanning-electron micrograph (SEM). **b**, **c**, **d**, Energy-dispersive X-ray (EDX) maps of the elements Pt, Zn, and O. The distributions of Pt (see panel **b**) and Zn (see panel **c**) are spatially well separated. Zn is only detected on the right-hand side of panel **c**. In this region, the ZnO structure is visible in the SEM in panel **a**. Oxygen is observed for both, the ZnO region as well as for the glass substrate (SiO_2) on which the Pt wire and the ZnO have been laser-printed.

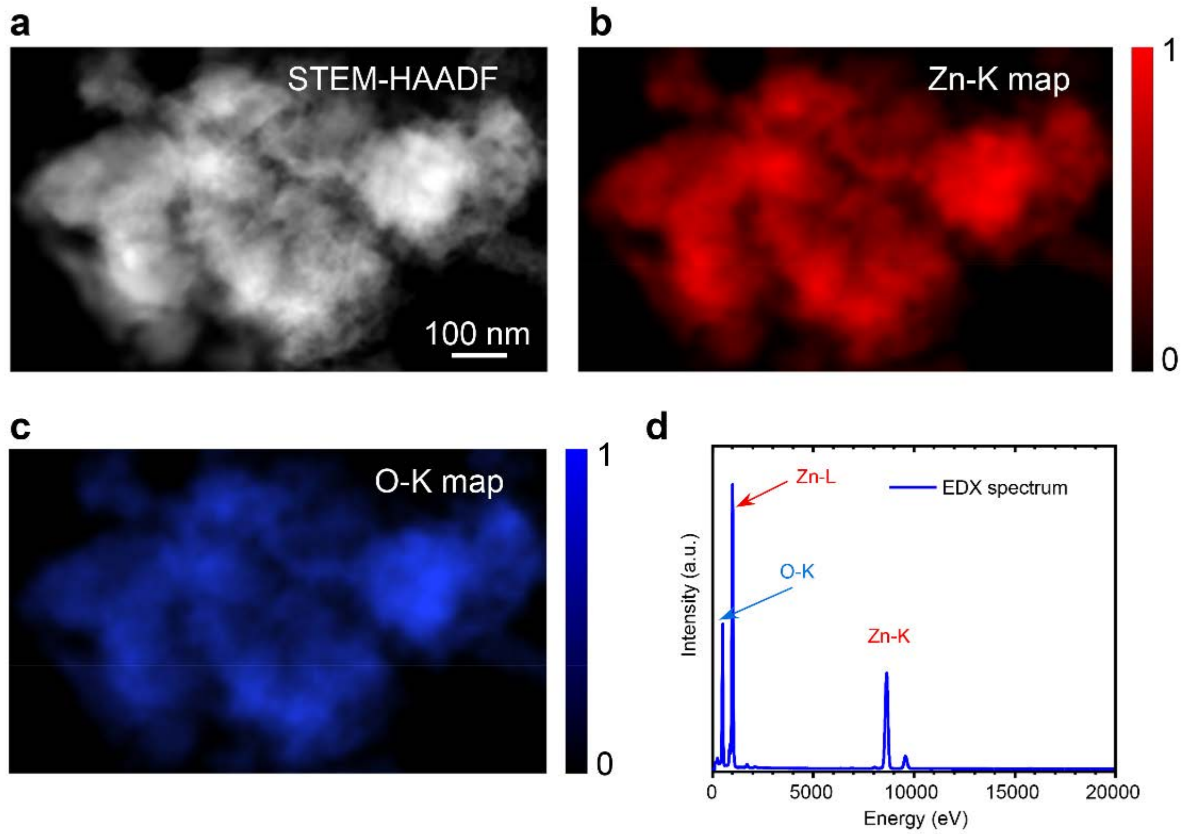

Figure S8: EDX spectrum of ZnO fragments. a, High-angle annular dark-field imaging (HAADF) from a STEM. **b** and **c,** EDX map of Zn and O. **d**, The EDX spectrum reveals that the main elements of the laser-printed structure are Zn and O. In this measurement, ZnO is first printed on a large footprint of 600 μm × 600 μm on a glass substrate. Next, the ZnO is scratched into fragments and transferred to a standard copper grid for further imaging and EDX spectrum acquisition.

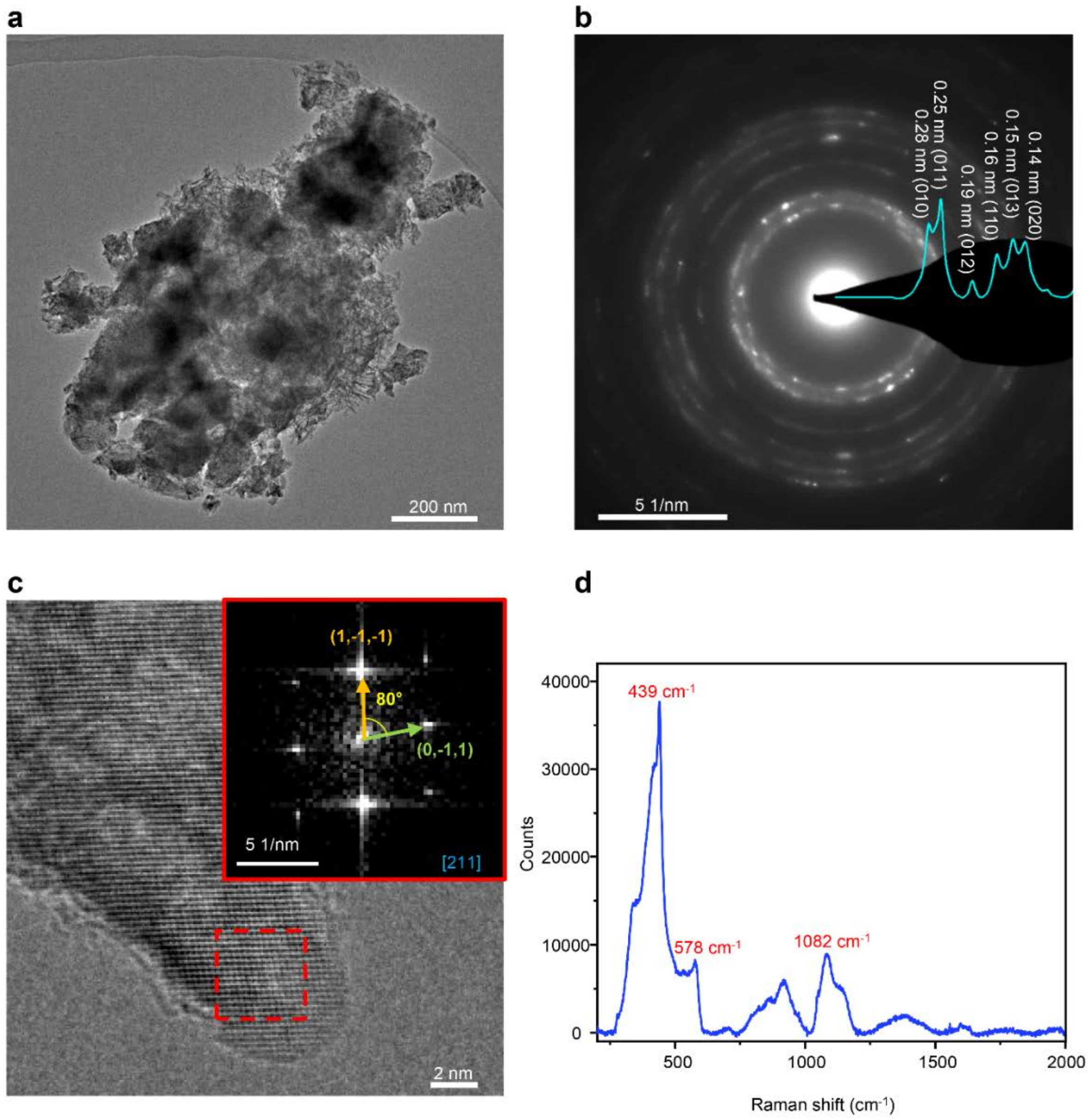

Figure S9: High-resolution TEM and Raman spectrum of laser-printed ZnO. a, TEM image of a ZnO fragment. **b**, Selected-area electron diffraction (SAED) with the profile of the summed signal (light blue) along the radial direction. **c**, High-resolution TEM (HRTEM) data. The inset shows the two-dimensional FFT (Fast Fourier Transform) of the area in **c** marked by the dashed red square. **d**, Raman spectrum of the laser-printed ZnO structure.

SEM-EDS mapping of a typical ZnO bump printed above a Pt wire confirms the presence of Zn and Pt in the designed locations (Fig. S7). EDX mapping of ZnO fragments from the printed area further confirms the presence of Zn at the expected energies of 0.997 keV and 8.647 keV, and O at 0.507 keV respectively, with no significant cross-contamination (Fig. S8). Furthermore, the selected-area electron diffraction (SAED) pattern (Fig. S9a, b) correspond to the diffraction rings in the SAED that can be perfectly indexed by the planes from hexagonally closed packed (hcp) ZnO crystallites. FFT (Fast Fourier Transform) from HRTEM can also be indexed by hcp ZnO along the [211] direction (Fig. S9c). The resonance at 439 cm^{-1} in the Raman spectrum shown in Fig. S9d is assigned to the E_2^{high} mode, the resonance around 578 cm^{-1} to the A_1(LO) mode, and the resonance at 1082 cm^{-1} to the A_1(TO+LO) mode of ZnO[1,2].

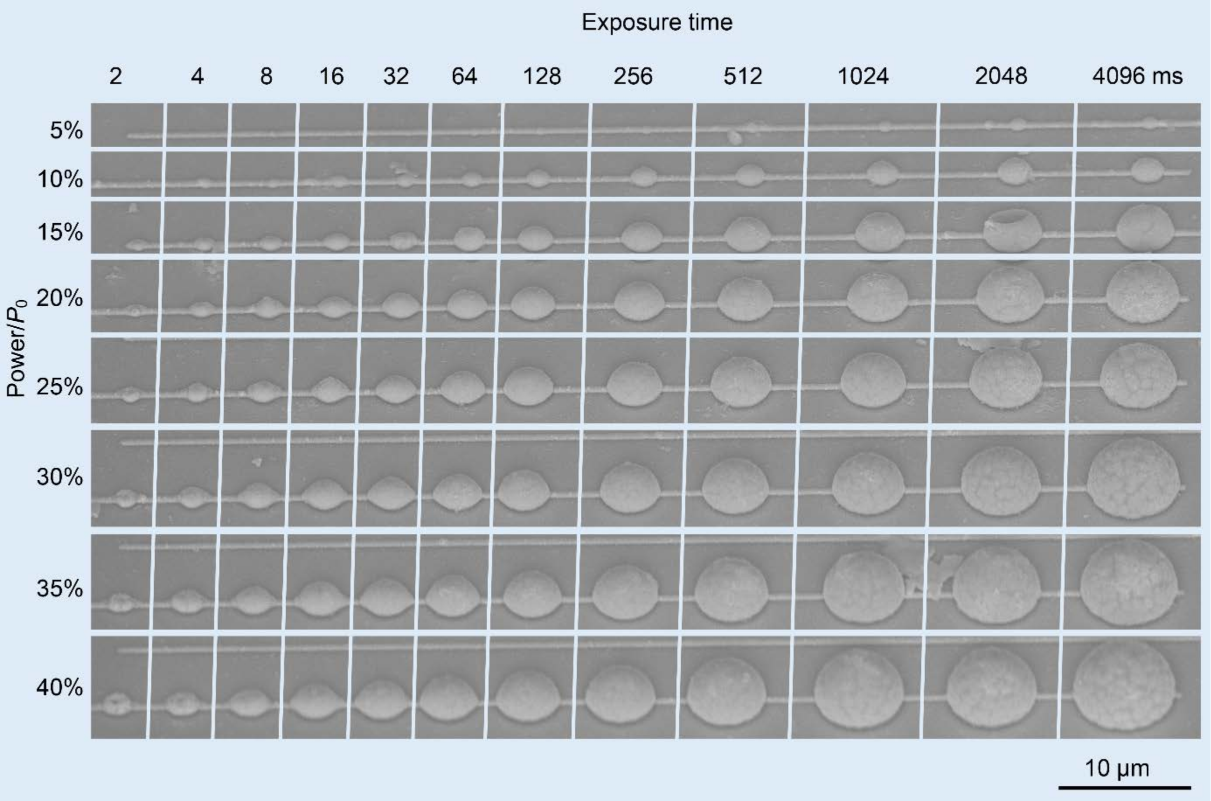

Figure S10: Oblique-view (45 degrees) SEM of ZnO hemispheres from point exposures. A 532 nm cw laser is focused onto a fixed position of previously printed Pt wires for a timespan as indicated at the top. In each column of the resulting "matrix", the exposure time is fixed and the laser power is increased from top to bottom as indicated on the left-hand side. These data show that the geometrical size of laser-printed ZnO structures can be controlled from hundreds of nanometers to beyond 10 µm by varying laser power and exposure time.

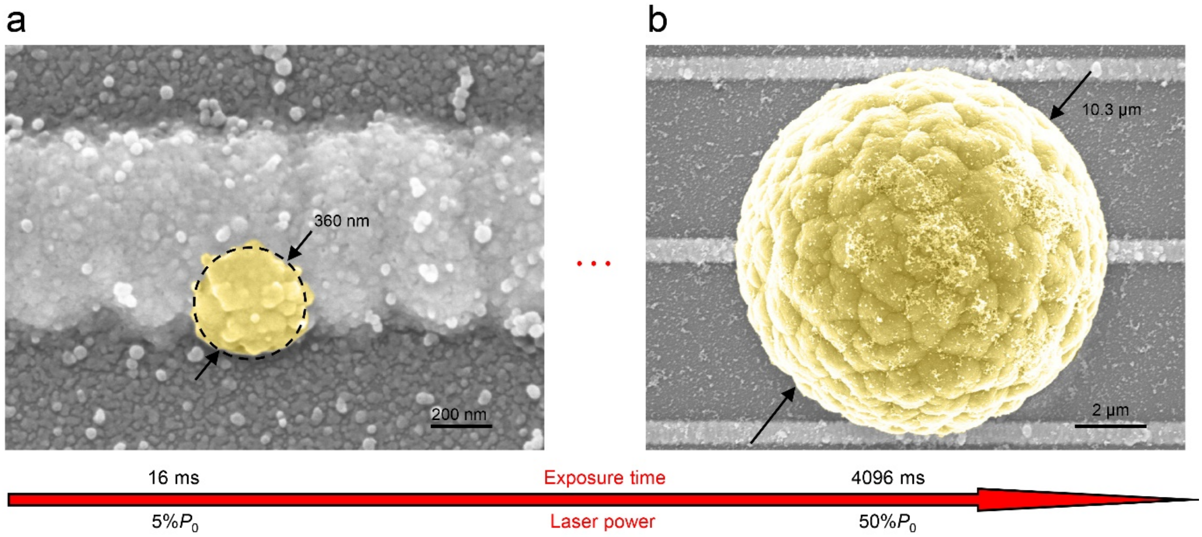

Figure S11: SEM of typical ZnO structures printed at low and high exposure dose. a, A ZnO hemisphere with a diameter of about 360 nm is achieved at a point exposure at P/P_0 = 5% and an exposure time of 16 ms. Note that this size is smaller than the linewidth of the Pt wire underneath the ZnO hemisphere. **b**, A ZnO hemisphere with a diameter of 10.3 μm results at P/P_0 = 50% and 4096 ms exposure time.

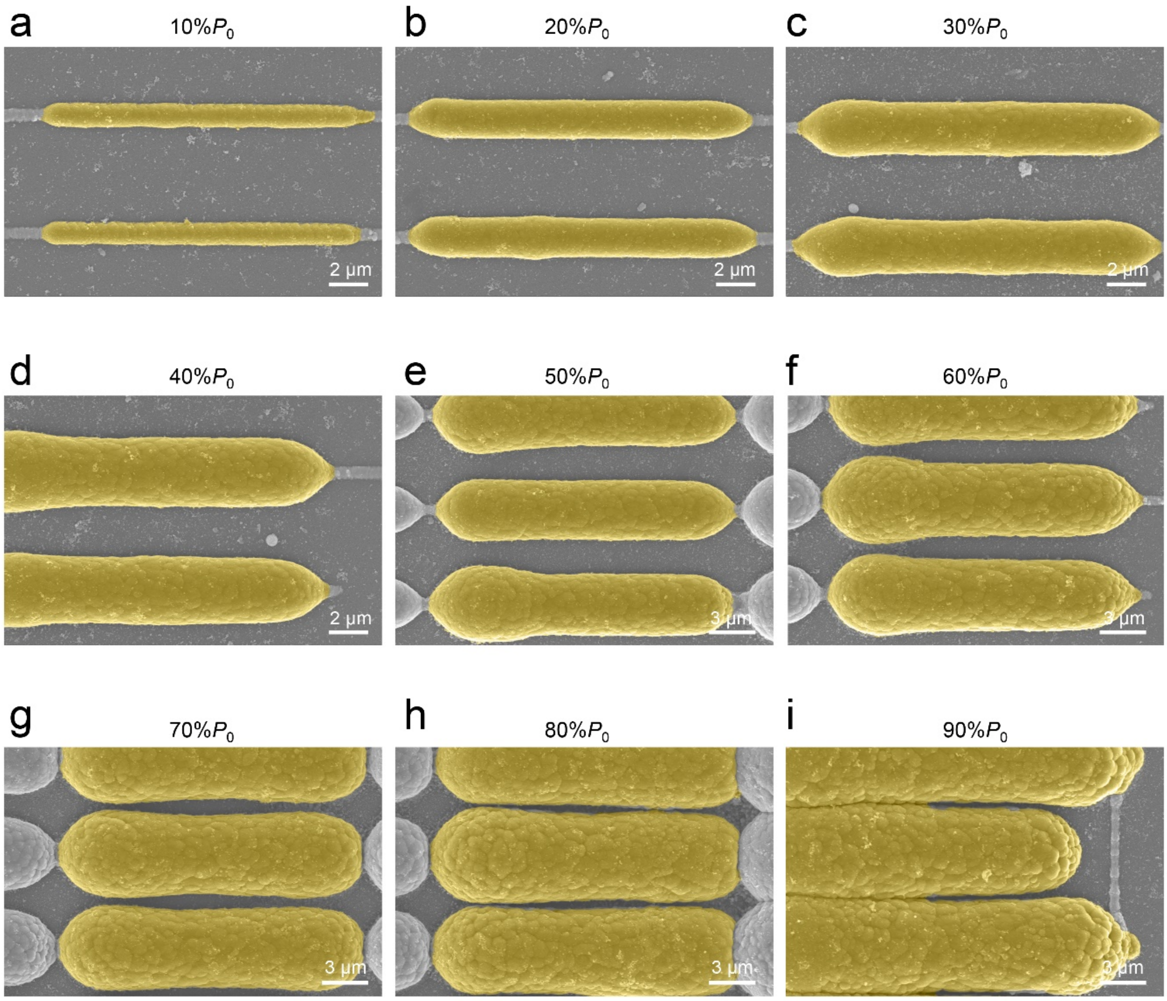

Figure S12: SEM of laser-printed ZnO lines versus laser power. To fabricate the ZnO lines, the laser focus is scanned perpendicular to the Pt wire repeatedly with a period of 0.5 μm. The focus speed is fixed at 20 μm/s, while the laser power P increases from 10%P_0 to 90%P_0, as indicated. The corresponding ZnO linewidth increases from **a**, 1.2 μm, to **b**, 2.0 μm, **c**, 2.8 μm, **d**, 3.5 μm, **e**, 3.9 μm, **f**, 4.8 μm, **g**, 5.3 μm, **h**, 5.6 μm, and to **i**, 6.0 μm.

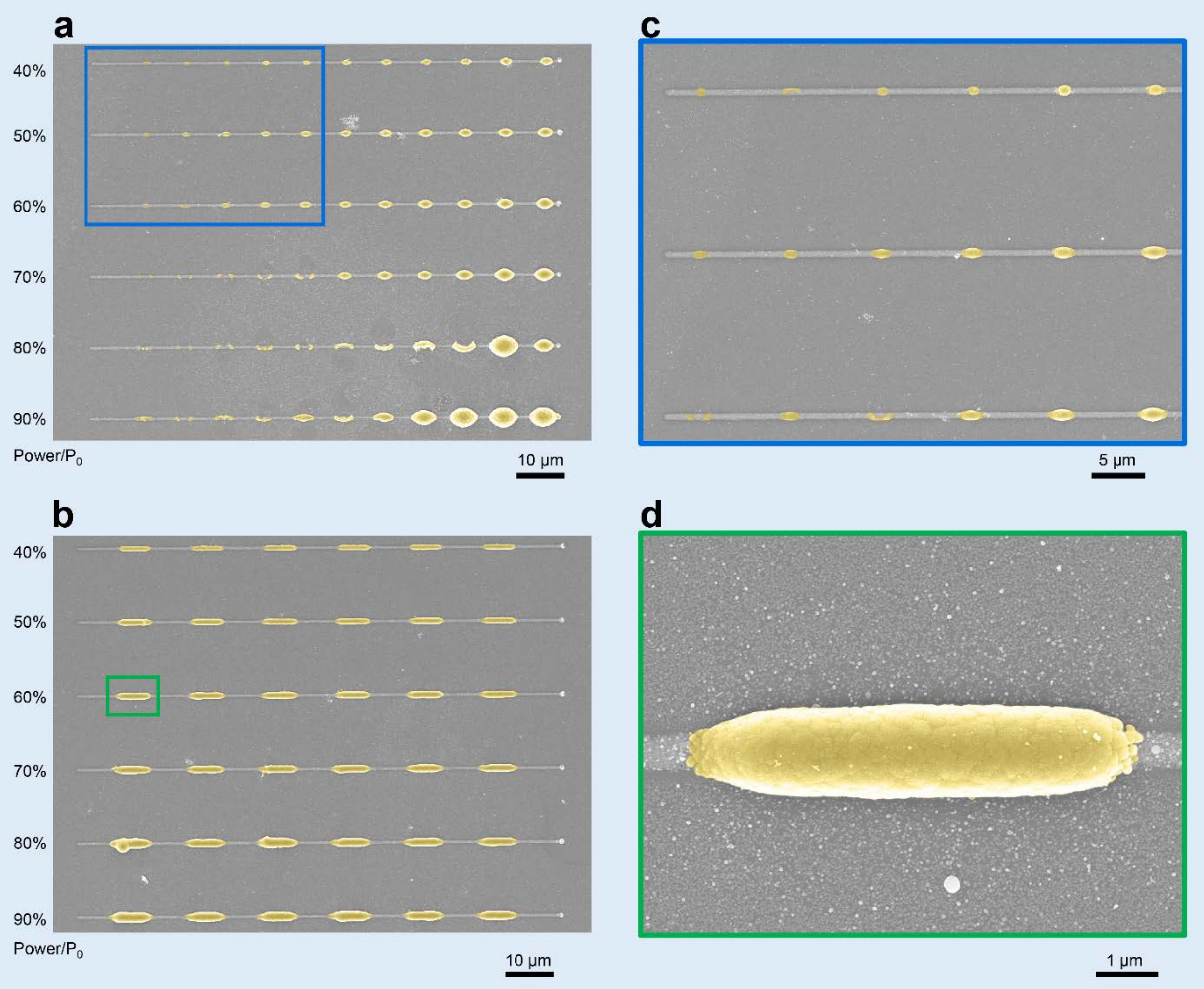

Figure S13: ZnO laser-printing on Ag wires. a, ZnO hemispheres printed on silver (instead of platinum) wires by point exposures. The Ag wires are rougher than the Pt wires. The laser power P increases from 40% to 90% of P_0 from top to bottom, and the exposure time increases from 2 ms to 4096 ms. **b**, ZnO lines printed on silver wires by increasing P from 40% to 90% of P_0. **c** and **d** are magnified SEM images of the areas marked in **a** and **b**, respectively.

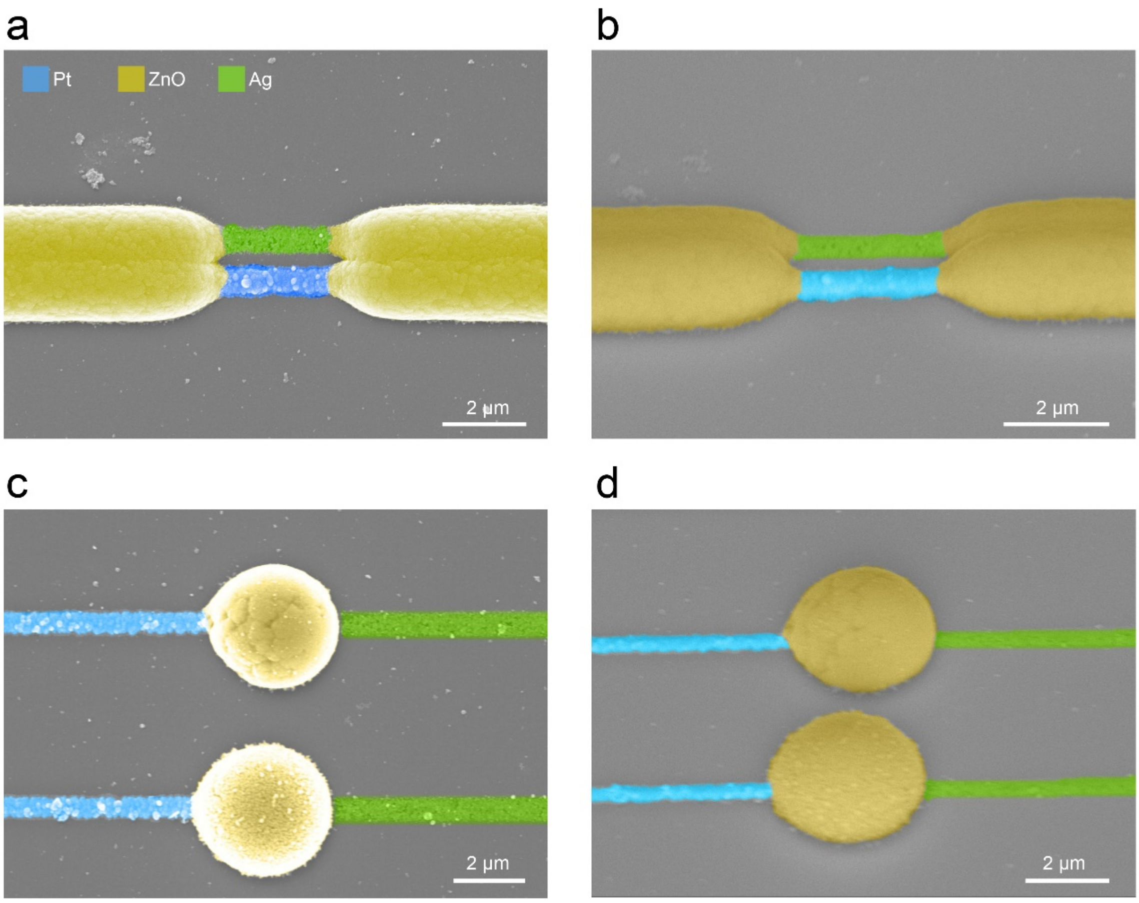

Figure S14: Gallery of laser-printed multi-material microstructures. a, SEM of parallel Pt and Ag wires covered with selectively laser-printed ZnO. The gap between Pt and Ag is about 350 nm wide. **b**, Oblique-view (45 degrees) SEM of the structure in **a**. **c**, SEM of a Pt-ZnO-Ag structure with the ZnO laser-printed over the gap in between the Pt and Ag wires. **d**, Oblique-view (45 degrees) SEM of the Pt-ZnO-Ag microstructure shown in **c**.

The multi-material structures in the main text are printed in the sequence Pt, ZnO, and Ag. Alternatively, Ag can also be printed before ZnO. The merit of this sequence is that the distance of the metal wires, which determines the channel length of diodes and transistors, can be precisely designed and controlled by the metal printing process, and is not determined by the geometrical size of the ZnO structures. In Fig. S14 a and b, Pt and Ag lines are printed sequentially with a center-to-center distance of 1 µm, leaving a gap of about 350 nm in between

them. In the next step, precise control of the ZnO printing parameters is not necessary as long as the ZnO layer covers the two metal lines. Likewise, in Fig. S14 c and d, Pt and Ag wires are aligned, with a gap of 1 μm between them. Subsequently, a 532 nm cw laser is focused onto the end of the Pt wire until a ZnO hemisphere grows and covers the gap between the Pt and the Ag wire.

One must be cautious though that, in this Pt-Ag-ZnO printing process, the laser-printed Ag wire composed of Ag nanoparticles can be slowly re-dissolved by the ZnO ink. This aspect limits the timespan over which Ag can be exposed to the ZnO ink. To minimize this issue, we have adjusted the pH value adequately (see main paper).

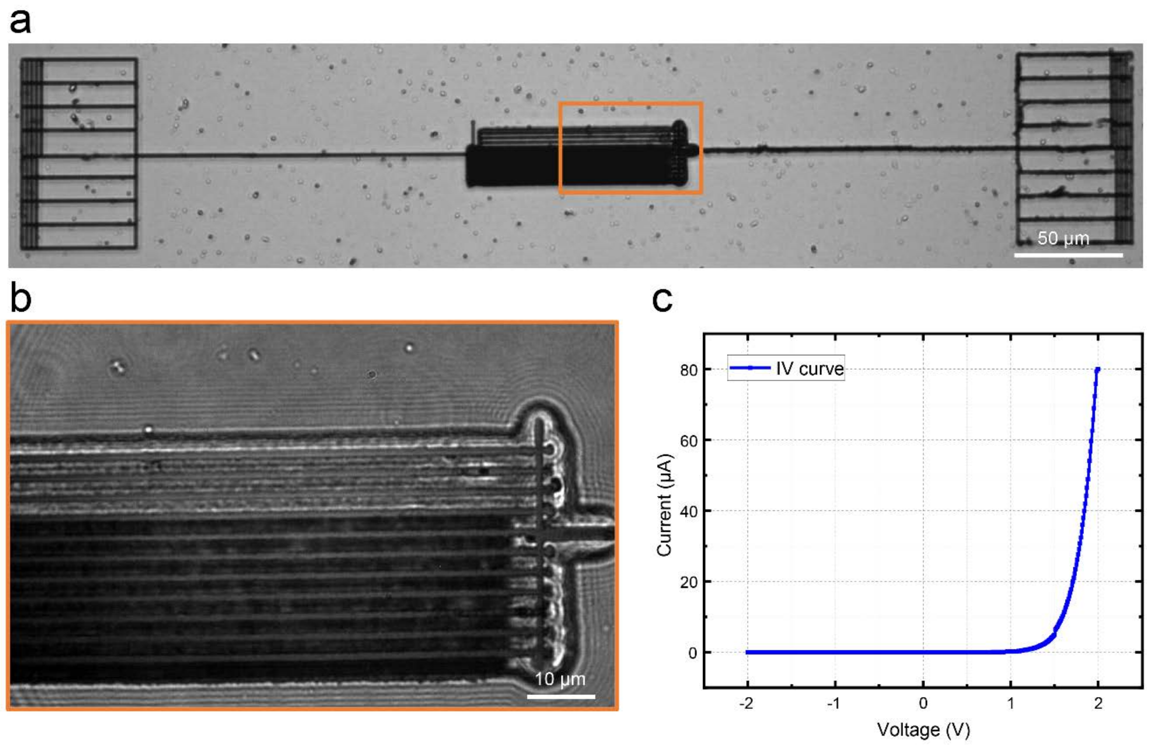

Figure S15: Sandwich (vertical) diode composed of Pt, ZnO, and Ag. a, Optical image of a completely laser-printed vertical diode with two large contact pads on the left and right. **b**, Magnified view of the marked area in **a**. **c**, Current-voltage characteristics of this diode.

In Fig. 3 of the main paper, a single diode and a planar diode with interdigitated electrodes are shown. Let us consider another example here to demonstrate the flexibility and versatility of the laser-printing approach. In the alternative approach shown in Fig. S15, Ag is laser-printed on top of the ZnO, forming a vertical or sandwich-mode diode. At the bottom, Pt wires with a period of 3 μm have been printed and connected to one laser printed grid-like electrode. Above these Pt wires, a layer of ZnO is printed at a laser-focus speed of 20 μm/s and a laser power of $P = 40\%P_0$. Due to the optical transparency of the laser-printed ZnO, the beam of a femtosecond laser is transmitted through the ZnO layer and can be focused onto the top surface of the ZnO. A Ag layer (black film in **a** and **b**) is subsequently deposited by scanning the laser focus. In this manner, we also manufacture the large-footprint grid-like electrodes in **a** for making electrical contacts in a probe station. The measured current-voltage characteristics is depicted in Fig. S15c.

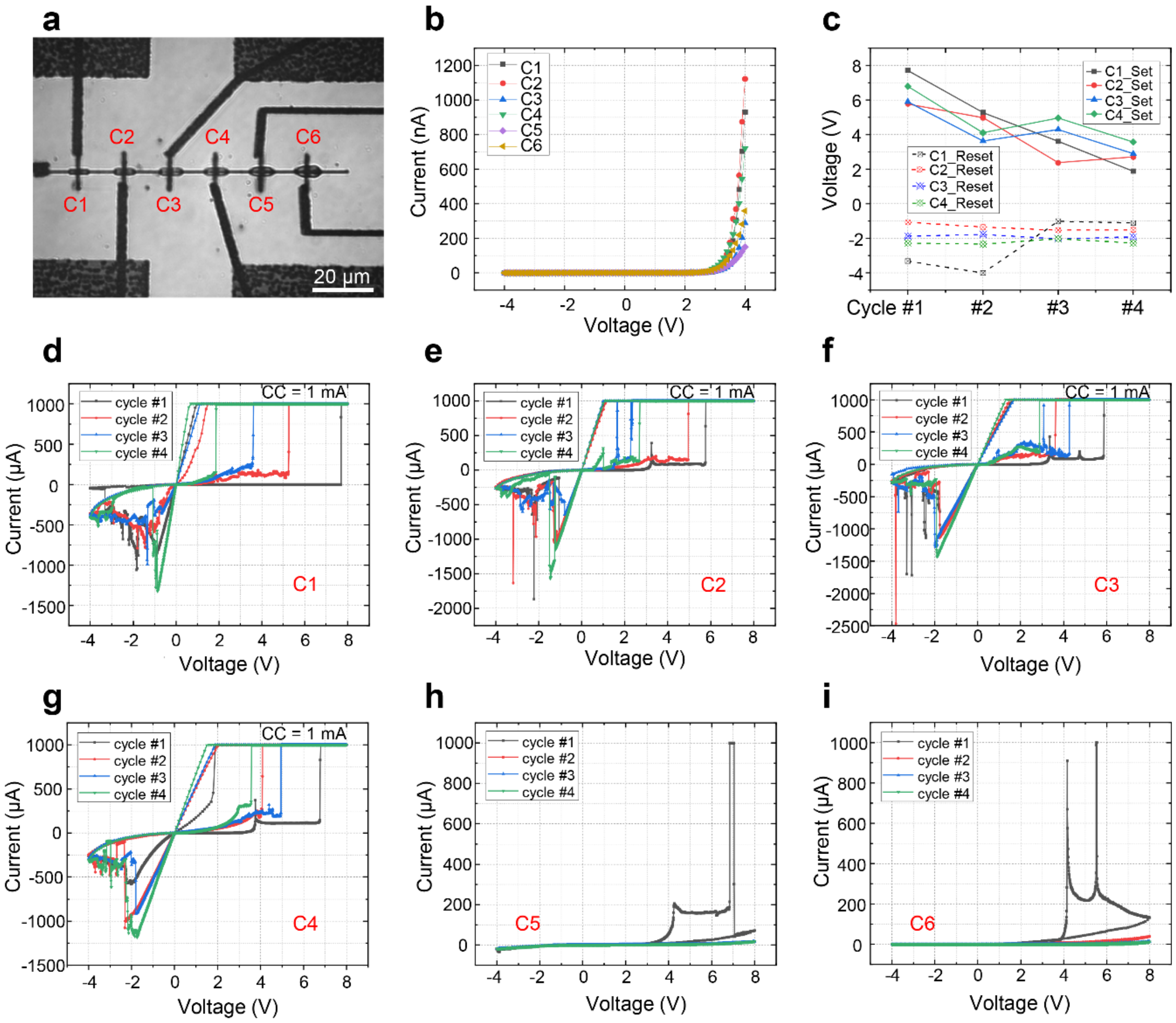

Figure S16: Changing the electrical performance of memristors in a 1×6 crossbar structures by controlling the ZnO layer thickness. a, Optical micrograph of a 1×6 crossbar structure. **b**, Current-voltage (I-V) characteristics of the 6 diodes in the crossbar before the "forming process" of the memristor. **c**, Set and reset voltages of 6 devices under 4 cycles of resistive switching. **d-i**, I-V curves of 6 devices under 4 full-sweep cycles.

All 6 ZnO bars have been fabricated on the same Pt wire with a period of 15 µm. The thickness of the ZnO bar increases gradually from C1 to C6, by controlling the laser power in the printing process. The laser powers *P* are 12%, 16%, 20%, 24%, 28%, and 32% of P_0 from C1 to C6. All the ZnO bars are connected to the 6 electrodes via Ag wires. Before complete resistive switching of the crossbar structures, I-V curves of all the structures are measured in a voltage range from

-4 to 4 V. A current rectifying behavior demonstrates two metal-semiconductor contacts. A large potential barrier for electrons forms at the Ag-ZnO interface, leading to a Schottky-like contact. A lower barrier height forms at the ZnO-Pt interface, leading to a more Ohmic-like contact. As the sweeping voltage is further increased to 8 V, we find that the crossbar structures C1-C4 can be switched between the low-resistance state (LRS) and the high-resistance state (HRS), as required for a memristor behavior. Devices C5 and C6 cannot be completely switched. We interpret this behavior as being due to a ZnO layer which is too thick for the formation of stable Ag filaments inside the ZnO. Compared with C5 and C6, the ZnO layers in C1-C4 are thinner, and Ag filaments are stably formed and ruptured in the voltage sweeping process. Moreover, after 4 cycles of voltage sweeping, we find that the set and reset voltages increase from C1 to C4 as shown in Fig. S16c. This behavior is consistent with the ZnO thickness increasing from C1 to C4. This overall behavior means that we can control the electronic performance (diode/memristor, set/reset voltage) by the ZnO thickness via the laser-printing parameters.

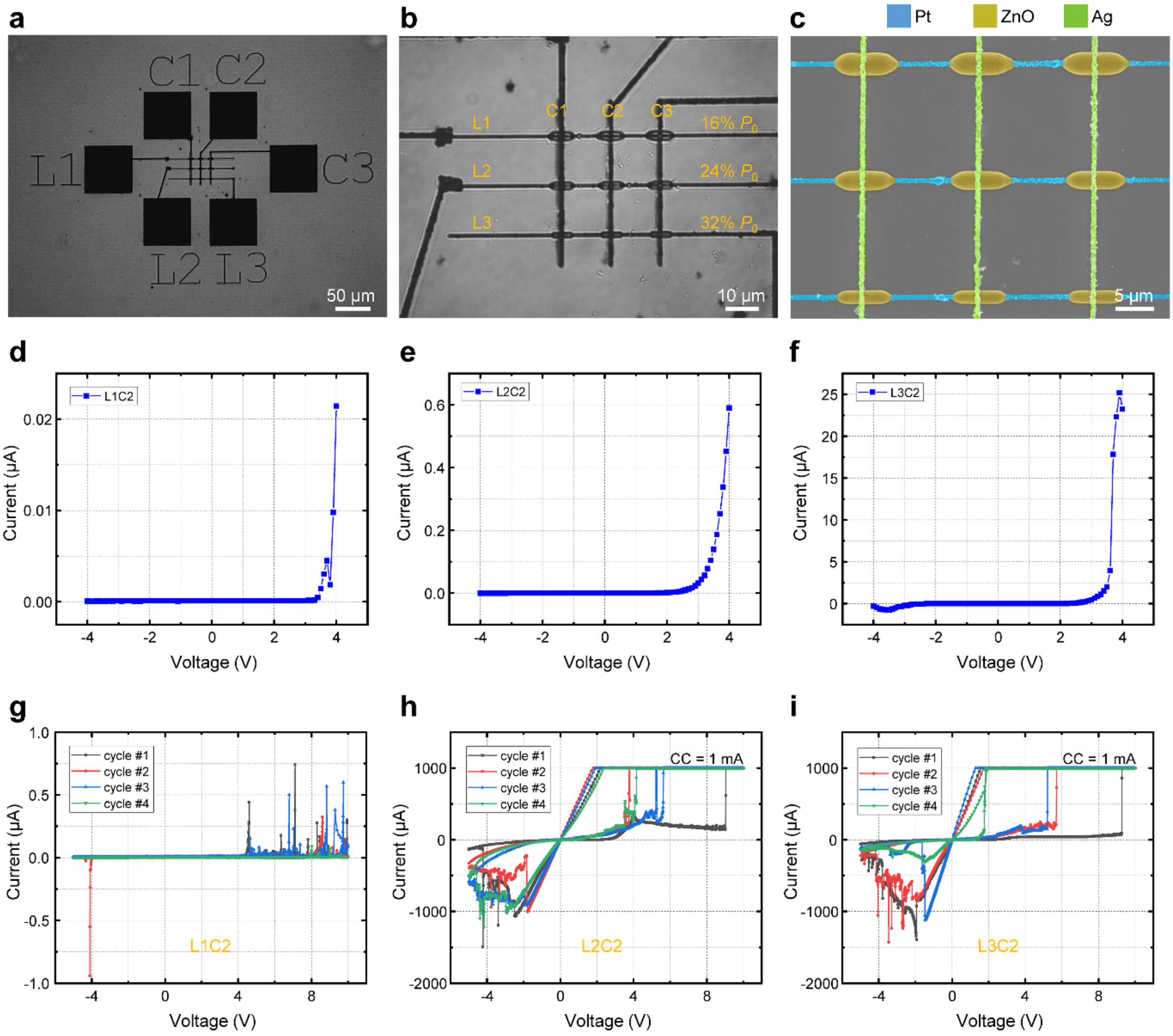

Figure S17: Electrical performance of 3×3 crossbar structures controlled by the ZnO layer thickness. a, Optical micrograph of a 3×3 crossbar structure with electrodes. **b** and **c** are zoomed-in optical and SEM images of the core of the 3×3 crossbar array. **d-f**, I-V characteristics of the 3 devices in the second column (L1C2, L2C2, L3C2) before the memristor formation process upon sweeping the applied voltage between -4 and 4 V. **g-i**, I-V curves of the same crossbar devices under 4 full-sweep cycles between -4 and 10 V.

The three lines in Fig. S17a and b are Pt wires connected to electrodes L1, L2, and L3, respectively. The three columns are Ag wires connected to the electrodes C1, C2, and C3, respectively. In each line, the thickness of the ZnO is fixed, while the thickness decreases from line L1 to line L3 (Fig. S17c). By sweeping the voltage from -4 to 4 V, an exponential increase

of the current is observed for all 3 crossbar devices in the second column. The current rectifying behavior reveals the same metal-semiconductor contact as in Fig. S16. A further increase of the voltage to 10 V leads to the complete formation of the memristors at the intersections L2C2 and L3C2. Resistive switching between the HRS and the LRS is observed. Compared with L2C2 and L3C2, L1C2 has a much larger ZnO thickness, which makes it impossible to work as a memristor inside of the 10 V sweeping-voltage range.

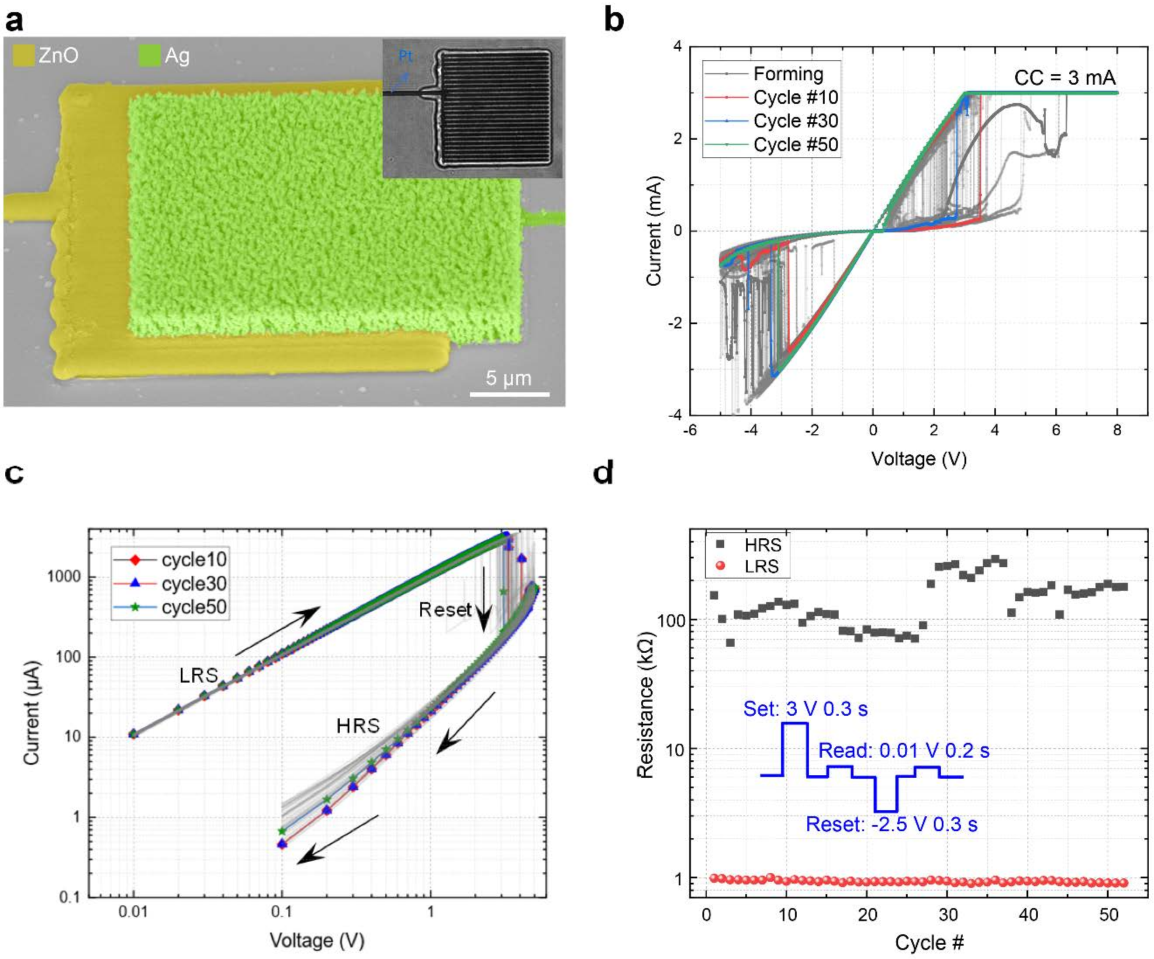

Figure S18: Sandwich-mode memristor. a, Colored SEM of a sandwich-mode memristor structure composed of a Pt electrode at the bottom layer, ZnO as the central isolating layer, and Ag on top. The inset shows an optical micrograph of a structure composed of Pt and a transparent ZnO layer before Ag laser-printing. **b**, Bipolar I-V curves taken during the formation process and during the cycle numbers #10 (red), #30 (blue), and #50 (green). All other of the 51 cycles total are shown in gray. **c**, I-V curves of the reset process from LRS to HRS plotted on a double-logarithmic scale. **d**, Endurance characterization of a sandwich-mode memristor.

Large-area sandwich mode memristors are completely laser-printed here. First, Pt wires with a period of 1 μm are printed using a 780 nm center-wavelength fs-laser to induce multi-photon reduction. Next, ZnO is laser-printed over the Pt wires at a laser power of P = 25% P_0 and a

focus speed of 20 μm/s. The inset in Fig. S18 clearly shows Pt wires below an optically transparent ZnO layer. In the next step, a Ag layer is laser-printed above the ZnO using the same fs-laser impinging from the ZnO side. To cover both the ZnO surface and the glass substrate, 7 layers of Ag are printed here, starting from the glass substrate to above the ZnO surface in steps of 0.5 μm.

Direct current (DC) voltage-sweep measurements show a bipolar resistive-switching behavior (Fig. S18b). For the set process (from the HRS to the LRS), the voltage is swept from 0 to 8.0 V and back to 0 V. For the reset process (from LRS to HRS), the voltage is swept from 0 to -5.0 V and back to 0 V. A compliance current (CC) of 3.0 mA is chosen to limit the maximum current and to protect the device from hard breakdown during the set process. The device has been measured for over 50 cycles. The corresponding I-V curves of the formation process and during cycle numbers #10, #30, and #50 are exhibited in Figure S18b.

The I-V characteristics of reset processes are also plotted on a double-logarithmic scale in Fig. S18c in order to reveal more details of the resistive switching. The rapid resistive switching, as evidenced by the steep slopes, is realized for all cycles. The I-V characteristics in the LRS, which exhibits a slope of about 1.00, are in good agreement with Ohmic conduction[3]. The high conductivity in the LRS indicates the formation of silver conducting filaments in the ZnO layer as illustrated in Fig. 4b. In contrast, the I-V characteristics of HRS exhibit varying slopes between ~1.9 to ~1.1. This evolution is attributed to the involvement of electrons from the metal electrodes and to thermally excited charged carriers[4].

The endurance characteristics of the device is investigated in the pulsed-voltage mode by switching the device between the HRS and the LRS in more than 50 cycles (Fig. S18d). A read-out voltage of 0.01 V is applied for a resistive state-test, which is sufficiently low to not affect the conductive filaments. The pulse voltages for set and reset process are 3V over 0.3 s and -

2.5 V over 0.3 s, respectively. Each voltage stimulation is followed by a resistance read-out pulse voltage of 0.01 V over 0.2 s. Again, the voltage is chosen to be sufficiently low to not affect the conductive filaments. The device shows low dispersion and high stability of both, the LRS and the HRS, during 52 full-sweep cycles, with an on/off ratio of ~10^2. The HRS possesses a resistance of about 10^5 Ω, while the LRS exhibits a resistance smaller than 10^3 Ω.

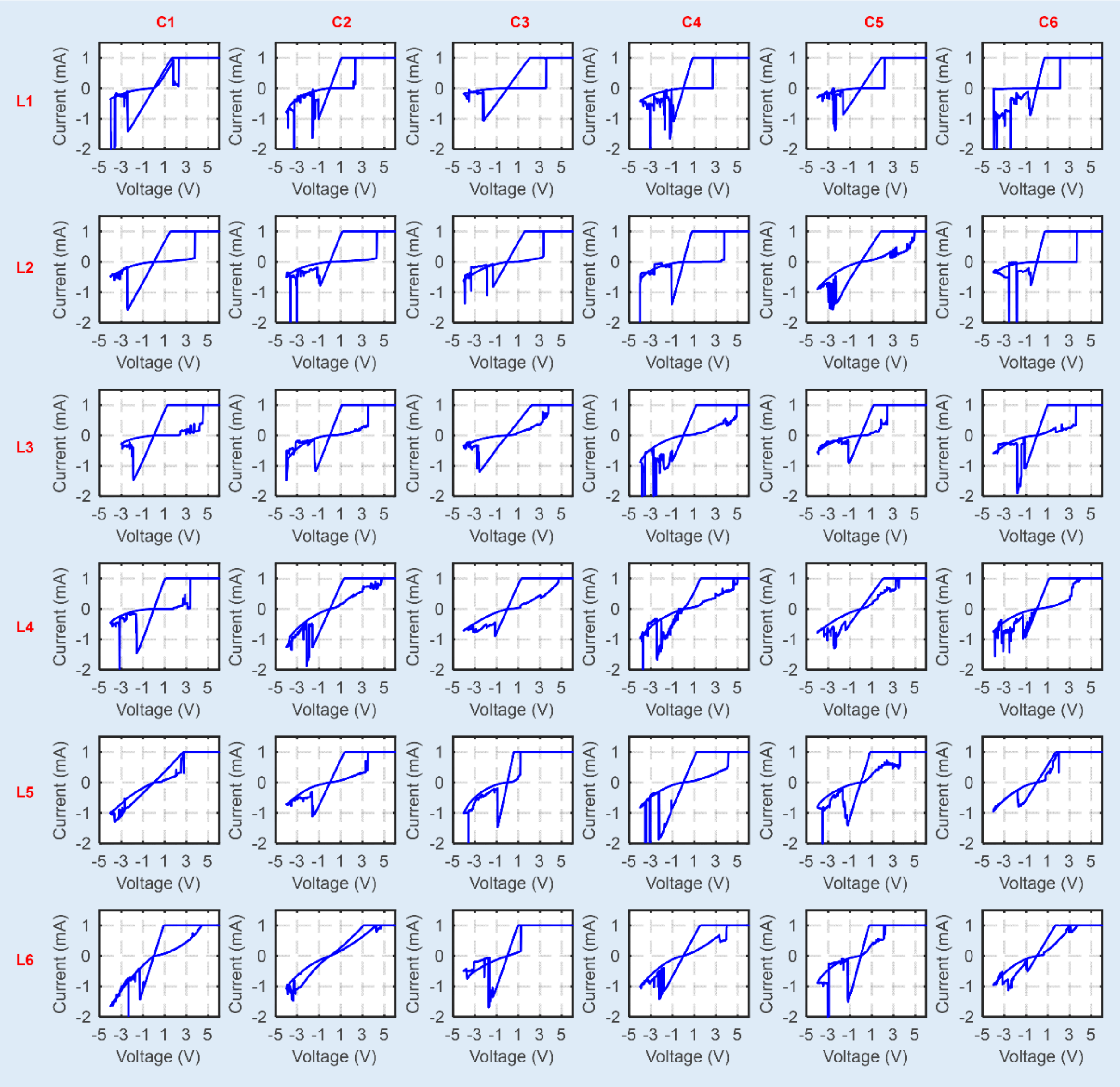

Figure S19: I-V Characteristics of all 6×6 memristors in a crossbar array. The line and column positions are marked in red. All memristors are set to the LRS below 5 V, and then reset from the LRS to the HRS by sweeping the voltage to -4 V.

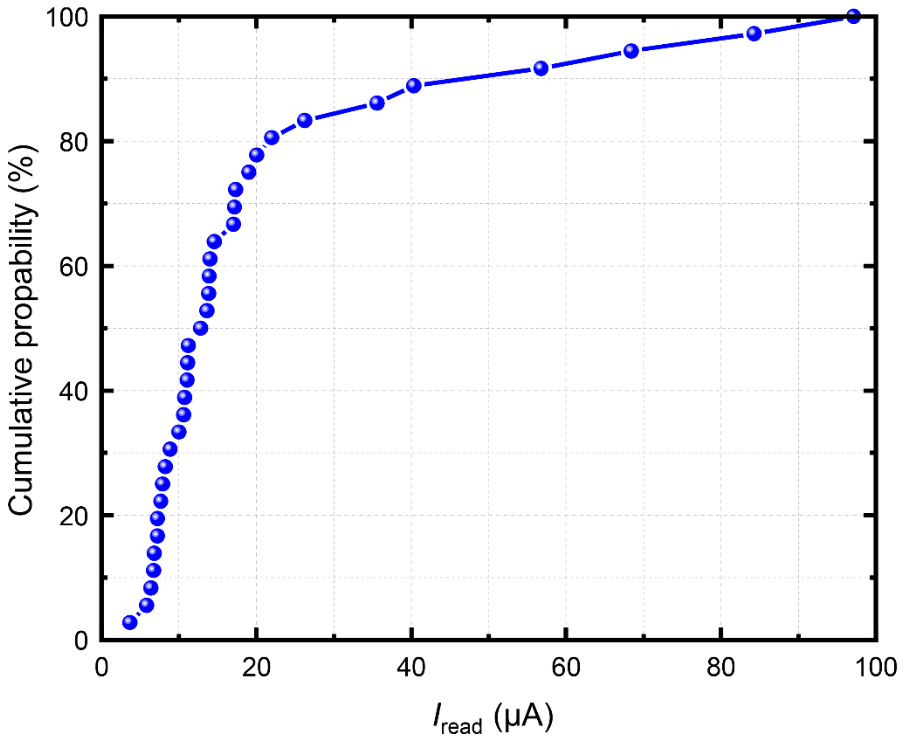

Figure S20: Empirical cumulative distribution of I_{read} of a 6×6 crossbar structure.

Empirical cumulative distribution of the obtained read-out currents of the memristors. The curve shows a log-normal distribution, which is common for memristor-based crossbar PUFs[5,6].

Hybrid laser- and inkjet-printed field-effect transistors

By combining laser-printing and inkjet printing, we have fabricated ZnO based electrolyte-gated field-effect transistors (EGTs). Laser-printed platinum and ZnO structures serve as drain and source electrodes and as a semiconducting accumulation-based channel, respectively (Fig. S21). The drains-source current is probed for sweeping the voltage from -5 V to 5 V. The expected exponential increase of the current versus voltage is observed. Furthermore, the I-V curve is symmetric with respect to positive/negative voltages, as expected from the symmetry of the Pt-ZnO-Pt structure.

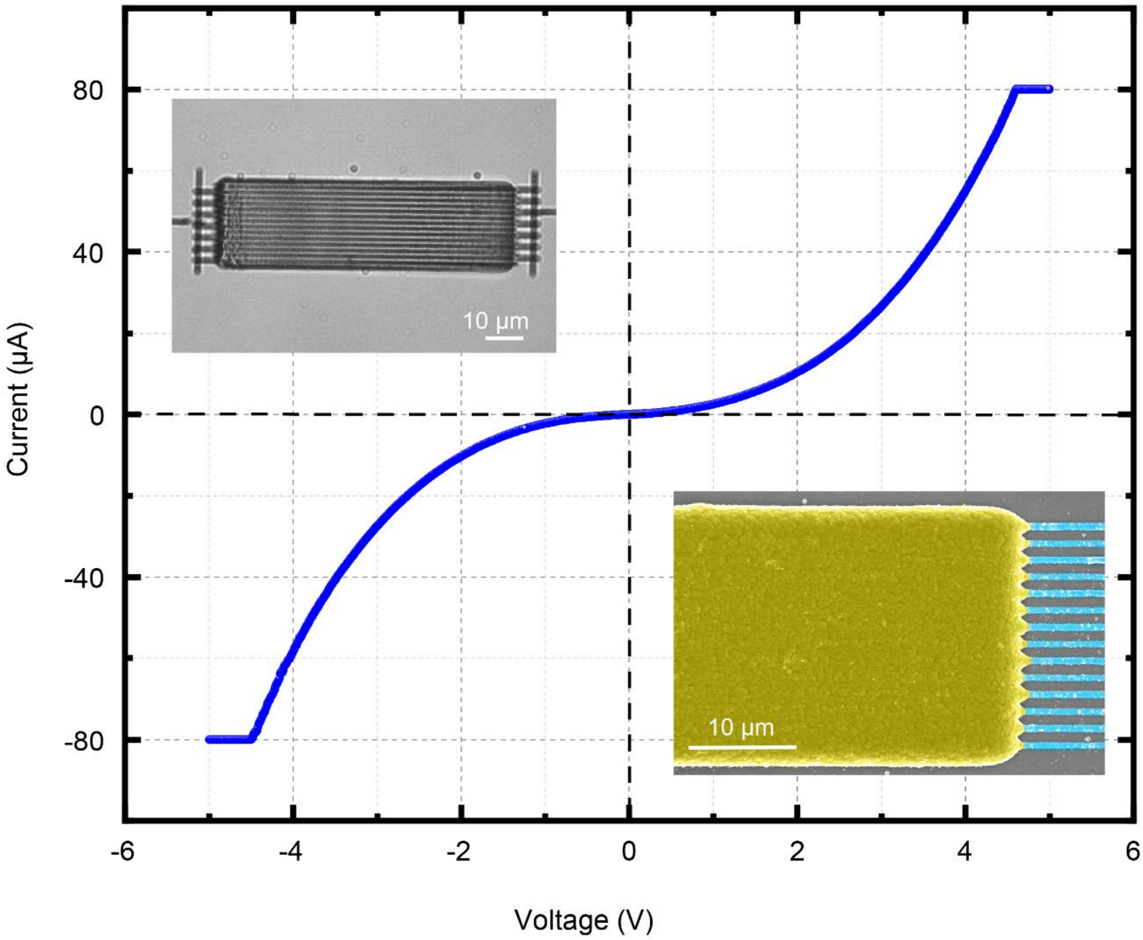

Figure S21: Current-voltage characteristics of a laser-printed Pt-ZnO-Pt structure before further inkjet printing. The compliance current is set to 80 µA. The insets show optical and SEM micrographs of the Pt wire array, with a layer of ZnO on top.

Starting from the laser-printed Pt-ZnO-Pt structure, the gate-dielectric with corresponding gate-electrode, and the silver contact pads, which are used to electrically connect the gate-, drain-, and source-electrodes, are all inkjet printed (Dimatix DMP 2831 inkjet printer) [7,8]. For the gate dielectric, a composite solid-polymer electrolyte (CSPE)[9,10] is used, which covers the entire channel area. Next, the conductive polymer PEDOT:PSS is inkjet-printed as the top gate-electrode on top of the CSPE, within the channel area. The composition of the whole transistor is schematically shown in Fig. S22a. Ordinary gate dielectrics might be compatible with the laser-printing process, but our choice for the CSPE is based on the fact that the CSPE provides a high gate-capacitance due to the formation of a Helmholtz-double layer at the gate/electrolyte and the electrolyte/channel interfaces, reducing the supply voltage to levels around 1 V. Figure S22b shows an optical micrograph with graphical representation of the hybrid laser- and inkjet-printed EGT. Here, the Pt linewidth is about 0.6 µm. The distance between the fingers, which determines the channel length (L), is about 1 µm. The channel width (W) is defined by the lateral overlap between one drain- and source-pair. This results in a W/L-ratio of 320.

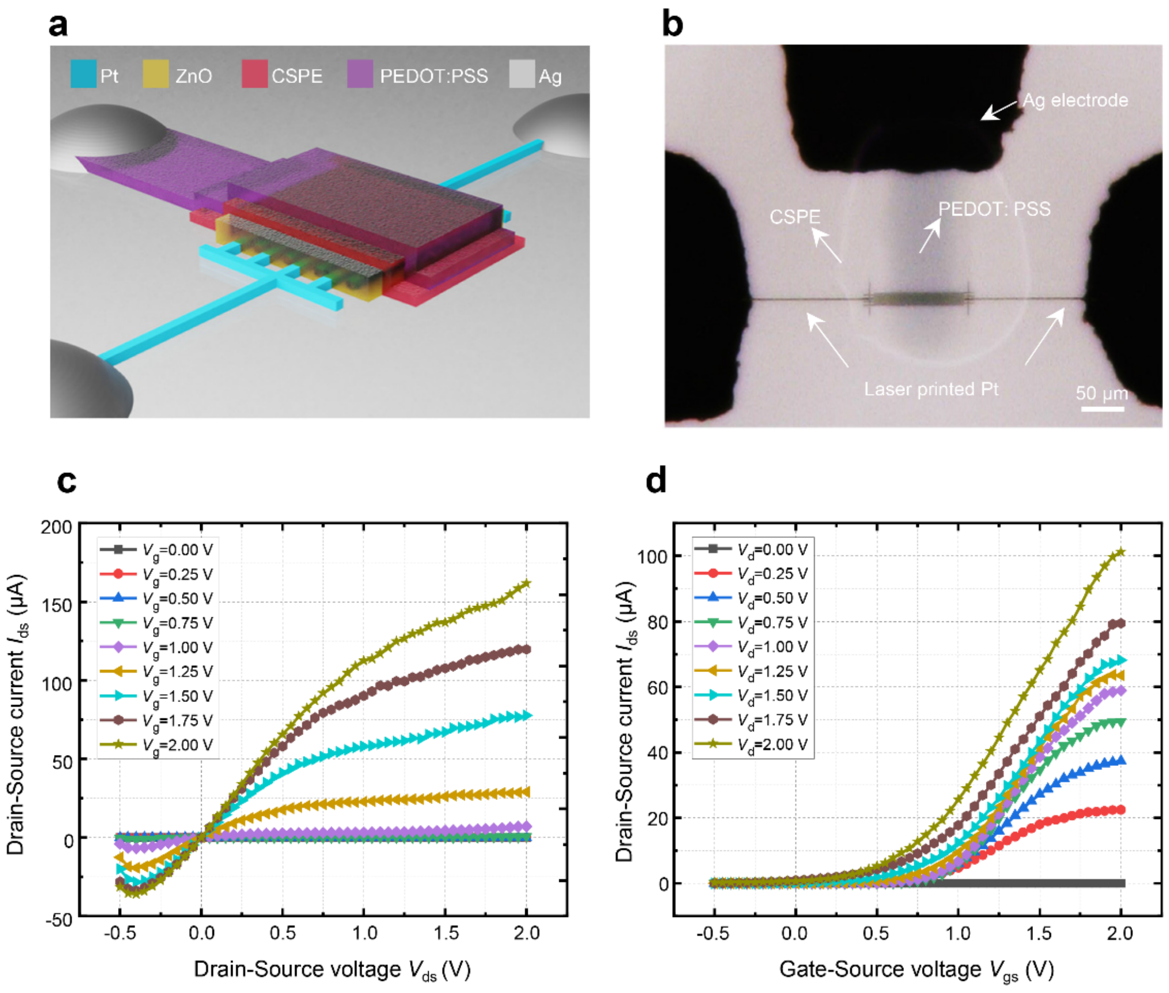

Figure S22: Transistor manufactured by combined laser- and inkjet-printing. a, Scheme of the transistor. Pt and ZnO are laser-printed on glass substrate. Above the ZnO, the electrolyte and the PEDOT:PSS layer are printed subsequently via ink-jet printing. Finally, Ag electrodes are inkjet-printed to connect drain, source, and gate. **b**, Optical micrograph of the final transistor. **c**, Drain current (I_{ds})-drain voltage (V_{ds}) curves of printed transistor in **b**. **d**, Transfer characteristics of the fully printed transistor.

The EGTs are measured and characterized at a relative humidity of around 50 %. From the transfer curve (see Fig. S22c), at a drain-source voltage (V_{ds}) of 2 V, the on/off-ratio results as 2.4×10^5, showing that the drain-source leakage currents are at a favorably low level of only several nA. Furthermore, the subthreshold slope (S) is 100 mV/dec and the threshold voltage (V_t) is around 800 mV, revealing that the EGT is operating as a normally-off transistor. In

addition, the gate-current does not exceed 20 nA. By the standards of electrolyte-gated transistors, these data represent a good behavior. It is an important factor in regard to the transistor power consumption. Finally, the output curves of the EGT show the typical transistor behavior (see Fig. 22d).

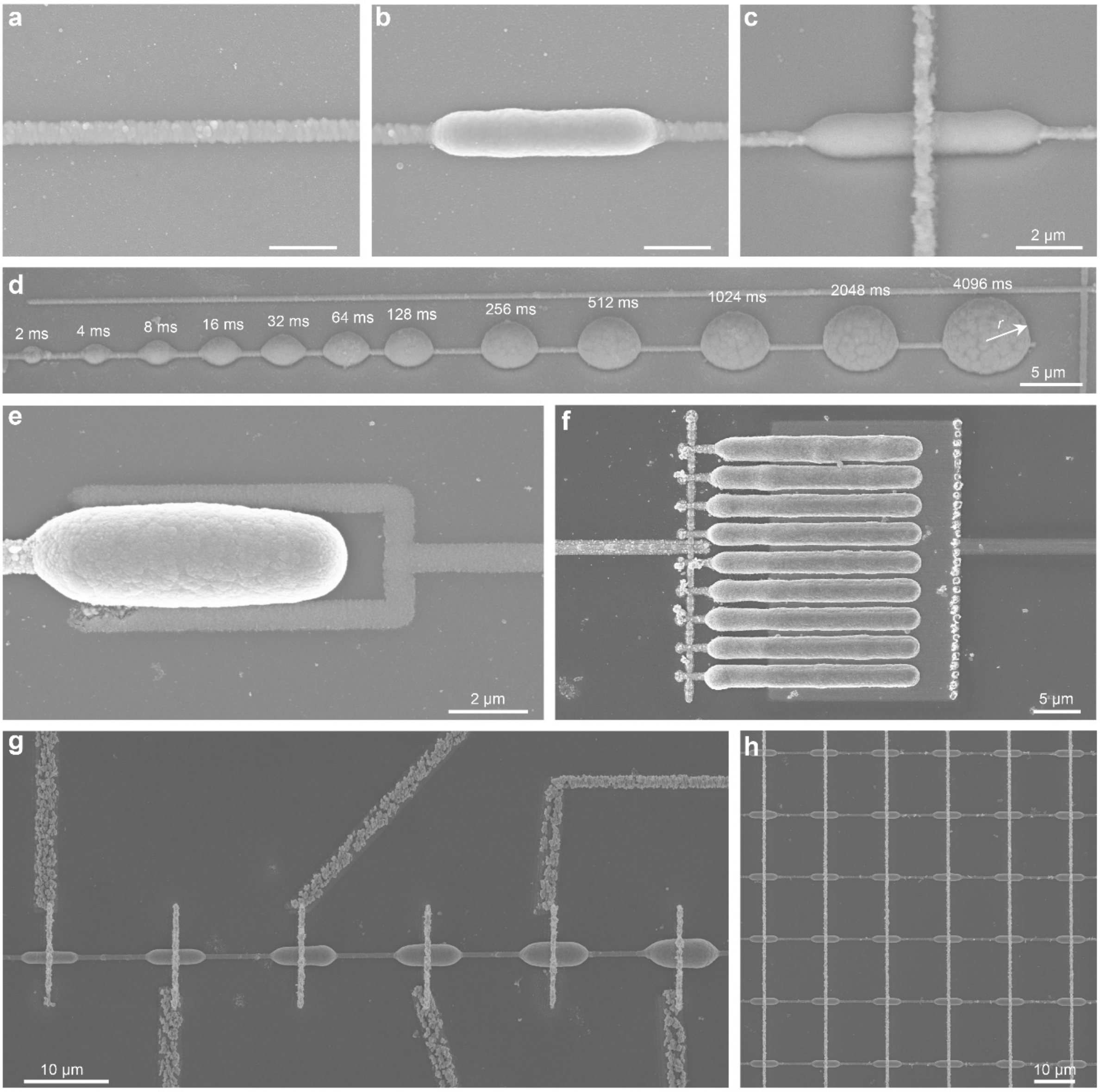

Figure S23: Not-colored versions of all electron micrographs of main paper.

a-c as Fig. 1 d-f, **d**, as Fig. 2a, **c** and **f**, as Fig. 3 a and c, **g**, as Fig. 4a. **h**, as Fig. 5c.

References

1 Liu, J. W. *et al.* 3D printing nano-architected semiconductors based on versatile and customizable metal-bound composite photoresins. *Adv. Mater. Technol.* **7**, 2101230 (2022).

2 Cusco, R. *et al.* Temperature dependence of raman scattering in ZnO. *Phys. Rev. B* **75**, 165202 (2007).

3 Chiu, F. C. A Review on conduction mechanisms in dielectric Films. *Adv. Mater. Sci. Eng.* **2014**, 578168 (2014).

4 Zhu, J. D., Zhang, T., Yang, Y. C. & Huang, R. A comprehensive review on emerging artificial neuromorphic devices. *Appl. Phys. Rev.* **7**, 011312 (2020).

5 Yoshimoto, Y., Katoh, Y., Ogasahara, S., Wei, Z. & Kouno, K. A ReRAM-based physically unclonable function with bit error rate < 0.5% after 10 years at 125°C for 40nm embedded application. *IEEE Symposium on VLSI Technology,* 1-2 (2016).

6 Liu, R., Wu, H., Pang, Y., Qian, H. & Yu S. Extending 1kb RRAM array from weak PUF to strong PUF by employment of SHA module. *Asian Hardware Oriented Security and Trust Symposium (AsianHOST)*, 67-72 (2017).

7 Marques, G. C. *et al.* Printed logic gates based on enhancement- and depletion-mode electrolyte-gated transistors. *IEEE T. Electron Dev.* **67**, 3146-3151 (2020).

8 Baby, T. T. et al. A general route toward complete room temperature processing of printed and high performance oxide electronics. *ACS Nano* **9**, 3075-3083 (2015).

9 Marques, G. C. et al. Influence of humidity on the performance of composite polymer electrolyte-gated field-effect transistors and circuits. *IEEE T. Electron Dev.* **66**, 2202-2207 (2019).

10 Nasr, B. *et al.* High-speed, low-voltage, and environmentally stable operation of electrochemically gated zinc oxide nanowire field-effect transistors. *Adv. Funct. Mater.* **23**, 1750-1758 (2013).
