# Supplementary material for: Laser printed microelectronics
Source: Nat Commun. 2023 Feb 27;14:1103. doi: 10.1038/s41467-023-36722-7 (PMC9968718; doi:10.1038/s41467-023-36722-7)
Supplement: Supplementary file 3 — Description of Additional Supplementary Files [file 41467_2023_36722_MOESM3_ESM.docx]

**Description of Additional Supplementary Files**

**File Name: Supplementary Movie 1
Description:** Smooth generation of transparent ZnO in the laser-printing process.

**File Name: Supplementary Movie 2
Description:** Re-dissolving of printed ZnO inside the ink at high pH value.
