# Supplementary material for: Laser printed microelectronics
Source: Nat Commun. 2023 Feb 27;14:1103. doi: 10.1038/s41467-023-36722-7 (PMC9968718; doi:10.1038/s41467-023-36722-7)
Supplement: Supplementary file 2 — Peer Review File [file 41467_2023_36722_MOESM2_ESM.pdf]

nature portfolio

Peer Review File

Laser Printed Microelectronics

Open Access This file is licensed under a Creative Commons Attribution 4.0 International License, which permits use, sharing, adaptation, distribution and reproduction in any medium or format, as long as you give appropriate credit to the original author(s) and the source, provide a link to the Creative Commons license, and indicate if changes were made. In the cases where the authors are anonymous, such as is the case for the reports of anonymous peer reviewers, author attribution should be to 'Anonymous Referee' followed by a clear attribution to the source work. The images or other third party material in this file are included in the article's Creative Commons license, unless indicated otherwise in a credit line to the material. If material is not included in the article's Creative Commons license and your intended use is not permitted by statutory regulation or exceeds the permitted use, you will need to obtain permission directly from the copyright holder. To view a copy of this license, visit http://creativecommons.org/licenses/by/4.0/.

REVIEWER COMMENTS

Reviewer #1 (Remarks to the Author):

The work presented by Wegener and co-workers reports a new route toward direct fabrication of microelectronics via direct laser writing. In particular, the relevant innovation here presented relies on laser induced hydrothermal deposition of ZnO (active semiconductor), over metal two photon printed metal lines. This clever coupling of two photon and single photon additive fabrication mechanisms allows for the realization of microelectronic circuits with very high resolution, substantially unachievable by other direct printing methods. The idea is clearly novel and the advancement of the state of the art substantial.

The authors present the main idea, with all the necessary characterization and theoretical and several different demonstrative implementations, including diodes, memristors, PUFs and also electrolytic gated transistors (the latter coupling the presented approach with more traditional inkjet printing step).

In general, the work is well written and easy to follow. The data are presented in coherent way and with well realized graphics. A lot of interesting supporting material is also provided alongside the main text.

I believe that the paper has not serious weakness.

In the following, I report some minor comments / suggestion that should be considered and addressed before eventual publication. In details:

In main text

- Which is the measurement error / accuracy of pH measurement? To understand the needed precision for achieving correct operation, since it seems that it is a very important parameter.

- At page 7 is reported that “The inset in Fig. 1e indicates that the ZnO structures are composed of nanoparticles with a mean size of ~27 nm.” Please indicate how the measure is done and the error / standard deviation of that measure.

- About diodes, given the peculiar structure of the presented diode and the resolution achieved, it could be very interesting to evaluate the achievable bandwidth (diodes can be used as rectifiers), and compare with similar devices built by additive manufacturing processes (see for example DOI: 10.1002/adma.202002329).

- About memresistive PUF crossbar device, the concept is very interesting and well presented. Nevertheless, I think the author should provide more characterization and info on environmental condition during experiments. In particular, the stability over time (and in function of the environmental humidity) should be discussed. A curve reporting the error rate variation over time,

over temperature and/or over humidity col improve a lot the demonstration. Even if this is just a proof of concept, the retention of the correct should be demonstrated over a significant time (days or weeks), or at least, strategies for overcoming the problem should be discussed (i.e. encapsulation with Parylene or other).

In supplementary

- The supplementary video 1 is not really convincing, since I think that it is not possible to see the ZnO growing. Please check.

- In supplementary Figure S2 (c,d), I guess the P/P0 should be in percentage. Moreover, P0 should be defined somewhere in the caption (even if specified in somewhere in the main text).

- More info on Thermal simulation should be reported

- In Fig S18b, why not all curves are reported? (maybe in grey as in the main text for analogue measurement). It could be useful since the peculiar behavior of the device.

Reviewer #2 (Remarks to the Author):

The authors present linked approaches to directly fabricate metal and ZnO structures via various direct laser writing processes. Probably it is the ZnO writing that is more interesting, as optical patterning of metals has been known for some time. While the ZnO writing appears to be unique, it does have the limitation that the mechanism is a hydrothermal process with the heat that drives the reaction being that generated by the interaction of the laser with a previously written metallic structure (so it is not possible to write ZnO structures on their own on a transparent substrate).

While the structures formed are reasonably interesting, and certainly different from anything previously published, I find it rather difficult to envision what real applications might result. While the properties of the deposited metals and ZnO are ok, they certainly do not match the properties of high quality materials such as formed via various vacuum deposition approaches (which could then be patterned via conventional methods). While the authors describe a few potential applications, e.g. security, I have my doubts a real market will emerge. Perhaps the situation would be different if the authors had demonstrated the deposition of high quality materials. In short, I would be much more positive if the authors had succeeded in the future work they describe in the conclusion “It is more than likely that inks for other semiconductor materials, e.g., In2O3 or SnO2 can be developed

along the lines of ZnO, too. The laser-printed semiconductors could also be used for optoelectronic devices, such as detectors or light-emitting diodes, that could be integrated with three dimensional optical circuitry, for which laser printing of polymer micro-optical devices has previously been shown to be a promising avenue."

Probably the authors can form polycrystalline In_2O_3 and SnO_2. However, the probability they can form "optoelectronic devices, such as detectors or light emitting diodes" with performance metrics anywhere near the performance metrics of current materials is quite low. Fundamentally, there is no reason the materials the authors form will be any better than materials currently formed by hydrothermal synthesis (and such materials generally have modest performance metrics due to various defects in their crystal structure inherent to the hydrothermal synthesis process).

I should add, the authors have formed and tested an impressive set of devices. It is quite surprising how far they can push the performance of their devices giving the modest quality of the materials they have to work with!

Finally, while the colourized SEM images are attractive, I suggest the original images be added to the SI as the colourizing makes it difficult to observe the details in the SEM images.

Reviewer #3 (Remarks to the Author):

The authors present a multi-material laser printing approach for metals and semiconductors with a resolution of approx. 1 µm. Their main invention is an ink for the thermal deposition of ZnO, while the two-photon reduction of metals has been established earlier. Yet, it is this invention that crucially lets the authors print a variety of microeletronic devices. Importantly, the inorganic materials are functional in their as-printed state and do not require a post-print heat treatment. In general, the work will be of significant interest to the field and also a broader audience. The author's methodology is sound and their conclusions are sufficiently supported with data.

I recommend acceptance of the manuscript after major revisions.

Below a few questions and suggestions to the authors.

Questions and comments to the authors:

1. It would be justified to mention the use of multiple inks and the sequential deposition process in the abstract—after reading the abstract, it was not clear to me that different inks were used, and I first thought the different materials were deposited from a single ink. The fact that multiple inks were used is important and should be briefly mentioned. Also, the washing steps between materials should be mentioned in the main text to paint the complete picture of the approach.

2. The authors don't show any geometry or material that cannot be done with conventional lithography-based processing. Can the authors briefly motivate the use of laser-printing for these kind of devices in the introduction? For the case of 3D architectures the advantage of two-photon lithography is exceptionally clear compared to lithography. For planar, three component electronics with moderate submicrometer resolution, the advantage over lithography is not directly apparent to the reviewer.

3. In general, the manuscript would be greatly elevated if the authors could show quantitative analysis of the electronic properties of the printed materials, as these are fundamental for high-performing microelectronics (band gap, conductivity). Could the authors please share conductivity data for printed lines and compare these to bulk conductivity or the conductivity of thin films? Along similar lines, the authors mention in-situ laser sintering of the metal lines (Page 3 bottom). It would be helpful to show proof of this sintering in the main text, that is, a high-resolution SEM image of the morphology of these lines (or, if available, a cross-section).

4. The authors highlight the fact that their materials are functional without a post-print heat treatment. I strongly agree that this is an important advantage. Nonetheless, I would suggest the authors argue in the introduction why they think the general sintering procedure is a crucial challenge to be avoided, so that the uninformed reader can appreciate this advantage?

5. The authors have missed some of the early work on metal printing by two-photon reduction and cite later work. I think they should merit the original, earlier work. (As a side note, I have extracted these references from a review article on high-resolution metal printing: Hirt, L., Reiser, A., Spolenak, R., Zambelli, T., Hirt, L., Spolenak, R., & Zambelli, T. (2017). Additive Manufacturing of Metal Structures at the Micrometer Scale. Advanced Materials, 29(17).)

a. References to be considered for Ag printing:

i. F. Stellacci, C. Bauer, T. Meyer-Friedrichsen, W. Wenseleers, V. Alain, S. Kuebler, S. Pond, Y. Zhang, S. Marder, J. W. Perry, Adv. Mater. 2002, 14, 194.

ii. T. Tanaka, A. Ishikawa, S. Kawata, Appl. Phys. Lett. 2006, 88, 81107.

iii. Y. Y. Cao, N. Takeyasu, T. Tanaka, X. M. Duan, S. Kawata, Small, 2009, 5, 1144.

iv. A. Ishikawa, T. Tanaka, S. Kawata, Appl. Phys. Lett. 2006, 89, 113102.

b. For two-photon deposition of Au and Cu:

i. W.-E. Lu, Y.-L. Zhang, M.-L. Zheng, Y.-P. Jia, J. Liu, X.-Z. Dong, Z.-S. Zhao, C.-B. Li, Y. Xia, T.-C. Ye, X.-M. Duan, Opt. Mater. Express 2013, 3, 1660.

ii. F. Stellacci, C. Bauer, T. Meyer-Friedrichsen, W. Wenseleers, V. Alain, S. Kuebler, S. Pond, Y. Zhang, S. Marder, J. W. Perry, Adv. Mater. 2002, 14, 194.

6. Just above Fig 2, in the main text, authors discuss the microstructure of ZnO and mention that the material is composed of ZnO nanoparticles. This terminology suggest an agglomeration of nanoparticles rather than a dense material. Is this the case? The analyses in the SI do not give a definitive answer to this question either. Along the same lines, it would be helpful to the reader if the authors could briefly mention the main conclusions from SI Figs 7-9 in the main text.

7. For the memristor applications, could the authors briefly mention to the uninformed reader why two different electrode materials (Pt and Ag) are required for this application? Is the use of two different metals crucial or would two Ag electrodes work as well?

8. For the memristor device, could the authors specifically state how the performance of the laser printed memristors compare to conventional devices? Is it comparable? The authors say their performance is of "high endurance" and "extremely stable", but for the reader from outside the memristor field, the performance is difficult to put into perspective. Please help the reader doing so.

9. How did the authors calibrate the printed thermocouples?

10. Could you mention with what range of substrates the process could be compatible other than glass? Does the substrate have to be transparent, or are there any other limitations? Could you deposit the ZnO on a metallic substrate or on an insulating substrate?

11. Out of curiosity: the fact that the authors use different wavelengths for the deposition of the semiconductor and the metals inevitably brings up the question of whether these materials could be selectively deposited from a single, mixed ink? Could the authors comment on this, either just as an answer to me as a reviewer, or, if the authors think it would be important information for the general readers, in the manuscript?

12. Two typos: Page 6 (page numbers and line numbers would be helpful), middle. Authors write: "A detailed parameter study has shown that the proper choice of the pH value of the ink is crucial to avoid resolution of the printed material by the ink itself after the laser exposure (see Supplementary Video 2)." I assume there is a typo, and "resolution" should be "dissolution"? Experimental section, ZnO ink, first line, "Zno" instead of "ZnO".

We should sincerely like to thank the reviewers for their careful reading and valuable comments. In what follows, *we repeat the comments of the three reviewers in red and italics*, we respond to them in green, we cite from the original manuscript in black, and we highlight changes made to the manuscript in blue.

Reviewer #1

The work presented by Wegener and co-workers reports a new route toward direct fabrication of microelectronics via direct laser writing. In particular, the relevant innovation here presented relies on laser induced hydrothermal deposition of ZnO (active semiconductor), over metal two photon printed metal lines. This clever coupling of two photon and single photon additive fabrication mechanisms allows for the realization of microelectronic circuits with very high resolution, substantially unachievable by other direct printing methods. The idea is clearly novel and the advancement of the state of the art substantial.

The authors present the main idea, with all the necessary characterization and theoretical and several different demonstrative implementations, including diodes, memristors, PUFs and also electrolytic gated transistors (the latter coupling the presented approach with more traditional inkjet printing step).

In general, the work is well written and easy to follow. The data are presented in coherent way and with well realized graphics. A lot of interesting supporting material is also provided alongside the main text.

I believe that the paper has not serious weakness.

We thank the reviewer for their detailed and very positive assessment of our work. Especially, they state that the "… idea is clearly novel and the advancement of the state of the art substantial …", "… work is well written …", concluding with "… the paper has not serious weakness.".

In the following, I report some minor comments / suggestion that should be considered and addressed before eventual publication. In details:

In main text

- Which is the measurement error / accuracy of pH measurement? To understand the needed precision for achieving correct operation, since it seems that it is a very important parameter.

This information was missing indeed. In the revised version of the manuscript, at the first occurrence of "pH value", we now state:

"… For the results presented in this article (unless indicated otherwise), we use a pH value of 9.86±0.01. The photothermally induced reaction …"

"… Quantitative investigation of the geometrical size of laser-printed ZnO by controlling P and Δt, for pH values of 9.86±0.01 and 10.00±0.02, respectively. …"

and further below:

"…Thereby the concentration of Zn2+ was fixed to 0.4 M and the pH value was adjusted to 9.86±0.01, 10.00±0.02 and 10.39±0.02, respectively. …"

In addition, we have provided further information in the Methods section:

"… The pH values were determined using a Mettler-Toledo Seven Easy pH meter equipped with a Mettler-Toledo InLAB Science Pro-ISM electrode (inside/outside electrolyte 3.00 mol L^{-1} KCl) equipped with a built-in temperature sensor (NTC). The built-in temperature compensation was used to arrive at the errors quoted in the main text. The electrode was calibrated by three-point calibration using pH 4.00, 7.00 and 10.00 calibrants (Roti®Calipure, Carl Roth). …"

- At page 7 is reported that "The inset in Fig. 1e indicates that the ZnO structures are composed of nanoparticles with a mean size of ~27 nm." Please indicate how the measure is done and the error / standard deviation of that measure.

We have revised the sentence under debate to:

"… printing step are shown in panels d-f of Fig. 1, other structures in Supplementary Figs. 5 and 6. The inset in Fig. 1e shows a magnified high-resolution electron micrograph. From 50 nanoparticles therein, we roughly estimate a mean ZnO nanoparticle size of 27±5 nm. We have performed further detailed …"

- About diodes, given the peculiar structure of the presented diode and the resolution achieved, it could be very interesting to evaluate the achievable bandwidth (diodes can be used as rectifiers), and compare with similar devices built by additive manufacturing processes (see for example DOI: 10.1002/adma.202002329).

All devices investigated have been measured in a probe station based on contacting to the structures by macroscopic metallic needles (see Methods). Such experiments will be very interesting for future studies but are beyond the scope of the present paper.

- About memresistive PUF crossbar device, the concept is very interesting and well presented. Nevertheless, I think the author should provide more characterization and info on environmental condition during experiments. In particular, the stability over time (and in function of the environmental humidity) should be discussed. A curve reporting the error rate variation over time, over temperature and/or over humidity col improve a lot the demonstration. Even if this is just a proof of concept, the retention of the correct should be demonstrated over a significant time (days or weeks), or at least, strategies for overcoming the problem should be discussed (i.e. encapsulation with Parylene or other).

All devices have been kept at and investigated under usual ambient laboratory conditions (no cleanroom). No measures for protection have been taken for any of the devices. Some of the devices were stored under ambient conditions for weeks before the measurements have been performed. Apart from that, we have not performed any systematic lifetime studies or aging analysis. We feel that such studies are far beyond the scope of our proof-of-principle experiments. Indeed, as the reviewer points out, the devices can be encapsulated (see below). To improve our paper in this regard, we have modified the Methods section as follows:

"… The large Ag electrode pads with footprints 70 µm × 70 µm shown in Figs. 4 and 5 were printed at a higher focus velocity of v=50 µm/s.

After completing the fabrication in an ordinary laboratory (no cleanroom environment), the devices were stored under ambient conditions in the laboratory. This included exposing them to large humidity in the summer time. In some cases, samples were stored for weeks before performing the characterization measurements. If necessary, the devices could be protected by standard encapsulating (e.g., parylene or expoxy) or even by a laser-printed polymer.

Material Characterization: Samples were characterized by a …"

In supplementary

- The supplementary video 1 is not really convincing, since I think that it is not possible to see the ZnO growing. Please check.

We felt we should share an example of such a movie with the reader because that is what the reader would see if they were to reproduce our experiments. One should be aware though that it is hard to see transparent materials such as ZnO in an optical image. Perhaps, the originally chosen video was not ideal. To improve the paper for the reader, we have prepared a different video in which an additional vertical metal line serves as a guide to the eye for the ZnO growth.

The new caption reads:

"… Supplementary **Video S1: Smooth generation of transparent ZnO in the laser-printing process.** Real-time movie. A Pt wire-array is immersed in the ZnO ink (pH 9.86). Next, a 532 nm wavelength continuous-wave laser is focused onto the Pt-substrate interface and scanned at a speed of 20 µm/s along the vertical direction, i.e., perpendicular to the Pt wires. A uniform and optically transparent ZnO block results. The vertical metal lines on the left-hand side provides a guide to the eye for the ZnO growth near the middle of the image.

…"

- In supplementary Figure S2 (c,d), I guess the P/P0 should be in percentage. Moreover, P0 should be defined somewhere in the caption (even if specified in somewhere in the main text).

The revised caption of Fig. S2 reads:

"**Figure S2: Temperature measured by laser-printed Pt-Ag thermocouples. a**, Photograph of a thermocouple sample. **b**, Optical image of 4 thermocouples with laser-printed Pt-Ag junctions. The inset shows a magnified view onto a Pt-Ag junction. **c**, Measured temperature increase under laser exposure in air environment at room temperature. The couples are exposed to a laser until steady-state is achieved, at which point the voltage is measured. The reference laser power is $P_0 = 21.5\ \mathrm{mW}$. **d**, Measured temperature increase under laser exposure in water environment at room temperature. …."

- More info on Thermal simulation should be reported

All parameters for the heat-conduction simulations have already been provided in Table S1. Beyond that, we believe that it serves the reader best if we provide the COMSOL files underlying these simulations. This is now done by modifying the caption of Figs. S3:

"**Figure S3: COMSOL simulations of temperature fields generated in the laser-printing process**. **a** corresponds to a bare platinum line, while b corresponds to a platinum line with a hemisphere of ZnO on top. Heat maps after 25 ms of exposure are shown, taken at P/P_0 = 20% and with a ZnO thickness of 1 µm in **b**. The heat maps within the yz-plane (parallel to metal line) and in the xz-plane (perpendicular to metal line) are shown one the left and right of each panel, respectively. The COMSOL files used for these temperature-field simulations as well as for those shown in Fig. S4 are provided via a link (see data availability section)."

"Data availability

The data underlying the figures within this paper, COMSOL files for thermal simulation, and Supplementary Information are published on the open-access data repository of the Karlsruhe Institute of Technology (https://doi.org/10.35097/794)."

- In Fig S18b, why not all curves are reported? (maybe in grey as in the main text for analogue measurement). It could be useful since the peculiar behavior of the device.

Following this suggestion, we have revised the figure. Herein, the additional curves are shown in gray.

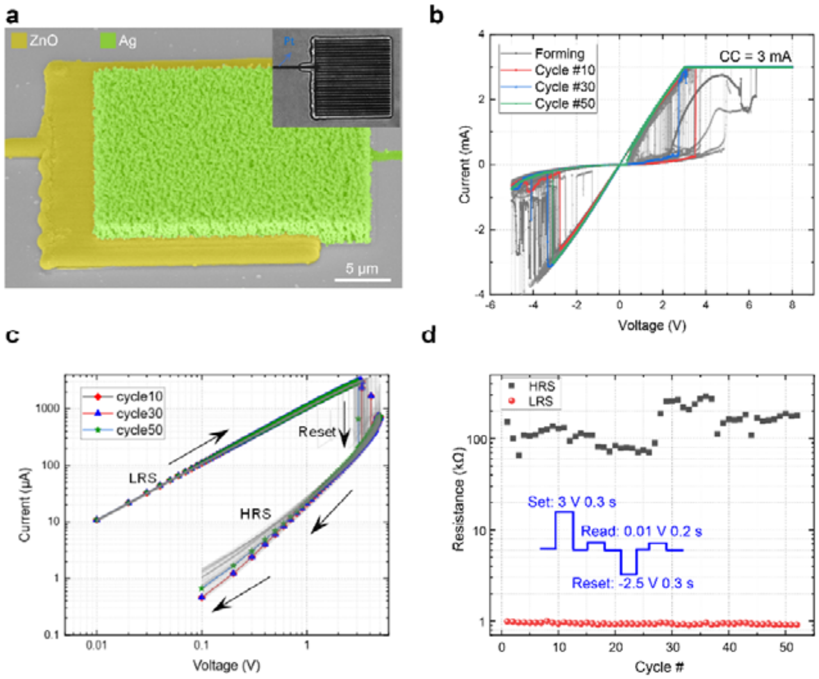

The new caption reads:

"… **Figure S18: Sandwich-mode memristor. a**, Colored SEM of a sandwich-mode memristor structure composed of a Pt electrode at the bottom layer, ZnO as the central isolating layer, and Ag on top. The inset shows an optical micrograph of a structure composed of Pt and a transparent ZnO layer before Ag laser-printing. **b**, Bipolar I-V curves taken during the formation process and during the cycle numbers #10 (red), #30 (blue), and #50 (green). All other of the 51 cycles total are shown in gray. **c**, I-V curves of the reset process from LRS to HRS plotted on a double-logarithmic scale. **d**, Endurance characterization of a sandwich-mode memristor.

…"

Reviewer #2

The authors present linked approaches to directly fabricate metal and ZnO structures via various direct laser writing processes. Probably it is the ZnO writing that is more interesting, as optical patterning of metals has been known for some time. While the ZnO writing appears to be unique, it does have the limitation that the mechanism is a hydrothermal process with the heat that drives the reaction being that generated by the interaction of the laser with a previously written metallic structure (so it is not possible to write ZnO structures on their own on a transparent substrate).

Indeed, the ZnO printing combined with metal printing without sintering is the main technological novelty underlying our present work.

Reviewer 2 is right by stating that sing local heating by absorption of light at the platinum wire only allows to print ZnO there, and not anywhere in 3D space. However, for electronics applications – and that is what the present paper focuses on – one neither wants nor needs ZnO *without* contact to a metal. In fact, one can consider the fact that the ZnO only grows on the metal as a serendipitous self-alignment process. This means that by nature our approach leads to good electrical contacts, which is a common challenge in many printed electronic devices and has been overcome in our approach. We kindly ask the reviewer to think about this. And yes, currently ".. it is not possible to write ZnO structures on their own on a transparent substrate", but that is neither wanted nor needed here.

We believe that this aspect is clearly explained by Figure 1 of the main paper and the corresponding manuscript text. Perhaps, the reviewer comes from a research direction for which such free-standing ZnO microstructures might be necessary.

While the structures formed are reasonably interesting, and certainly different from anything previously published, I find it rather difficult to envision what real applications might result. While the properties of the deposited metals and ZnO are ok, they certainly do not match the properties of high quality materials such as formed via various vacuum deposition approaches (which could then be patterned via conventional methods). While the authors describe a few potential applications, e.g. security, I have my doubts a real market will emerge. Perhaps the situation would be different if the authors had demonstrated the deposition of high quality materials. In short, I would be much more positive if the authors had succeeded in the future work they describe in the conclusion "It is more than likely that inks for other semiconductor materials, e.g., In2O3 or SnO2 can be developed along the lines of ZnO, too. The laser-printed semiconductors could also be used for optoelectronic devices, such as detectors or light-emitting diodes, that could be integrated with three dimensional optical

circuitry, for which laser printing of polymer micro-optical devices has previously been shown to be a promising avenue."

We thank the reviewer for stating that our structures are "… certainly different from anything previously published …". We believe that this is indeed true. This statement emphasizes that reviewer #2 does see the novelty of our work.

We agree with reviewer #2 in that the materials characteristics of the ZnO grown by us probably fall behind those of ZnO single crystals or ZnO deposited under ultrahigh-vacuum conditions and then structured by conventional lithographic means in a cleanroom. Nevertheless, we show in our paper an amazing variety of devices that simply work very well, especially including competitive memristor devices and arrays.

However, with all due respect, when reviewer #2 states that the properties of our materials "do not match the properties of high quality materials such as formed via various vacuum deposition approaches (which could then be patterned via conventional methods) ..." they seem to deny the advantages of the entire field of printed electronics (not only speaking of laser printing). The goal of this field is to manufacture electronic devices *without* high-vacuum equipment and *without* standard lithographic means, typically requiring a cleanroom facility and mostly rely on wafer scale processes. The structures we discuss are rather laser printed in a small setup by using a drop of a liquid ink. Thereafter, the devices are ready to use – without any further hidden pre- or post-processing and hence are especially interesting for small to medium numbers in lightweight electronic solutions for smart objects, wearables as well as internet of things applications.

In other words, our results on laser-printed devices should be compared with other approaches of solution processing. This is done in the abstract, the introduction, as well as in the conclusions. Our approach does not aim at competing with existing standard cleanroom processes (e.g., CMOS processes) but should rather be seen as an alternative or complementary route towards electronic devices and systems for applications in the security, wearable or internet of things sector.

Therefore, we also respectfully disagree with the reviewer in terms of the statement "I have my doubts a real market will emerge." The entire field of printed electronics believes in a market potential and also business analysts provide yearly forecasts for emerging printed electronics products (IDTechEx Market Research: https://www.idtechex.com/en/reports/printed-and-flexible-electronics/1). In particular, printed active electronic structures and small logic, memory or signal processing devices are needed in many emerging applications such as point-of-care medical diagnostics, security features integrated into smart objects, and many more.

Therefore, we do believe in future real market here and we will continue working in this direction. As a result, we have put speculations about yet other material systems that can likely be laser printed into our conclusions. Perhaps, this was *not* a convincing idea – as these are merely speculations. Therefore (also concerns next point), we have taken these speculations about the future completely off the conclusions.

The revised complete (shortened) conclusions read:

"… We provided a number of proof-of-principle demonstrations for direct laser printing of functional electronic devices and entire electronic circuits with minimum feature sizes down to <1 µm. A crucial prerequisite for this breakthrough has been the definition of an inorganic semiconductor (ZnO) ink for laser printing via photothermal synthesis. This ink and the associated printing modalities are conceptually distinct from both laser printing of polymers and metals. The advantages of direct laser printing of memristors and circuits based thereupon are the complete additive manufacturability, the

possibility of 2D and 3D integration in one process, and the flexibility to design each individual device according to the intended electrical behavior. This especially implies that no high-vacuum deposition and no standard cleanroom lithographic processes are needed for any of the devices presented in this work."

Probably the authors can form polycrystalline In2O3 and SnO2. However, the probability they can form "optoelectronic devices, such as detectors or light emitting diodes" with performance metrics anywhere near the performance metrics of current materials is quite low. Fundamentally, there is no reason the materials the authors form will be any better than materials currently formed by hydrothermal synthesis (and such materials generally have modest performance metrics due to various defects in their crystal structure inherent to the hydrothermal synthesis process).

As discussed above, we have removed the statement on In2O3 and SnO2 as well as all other speculations for future work from our conclusions (this is the only place where this occurred). Apart from that, we kindly ask reviewer #2 to think about the importance and impact of the new last sentence of the revised conclusions (see above).

I should add, the authors have formed and tested an impressive set of devices. It is quite surprising how far they can push the performance of their devices giving the modest quality of the materials they have to work with!

We understand that this statement, which ends with an exclamation mark, is meant as a complement. Regarding the "modest quality of the materials", we refer to our above discussion. It is simply not meaningful and helpful to compare our direct laser-printed materials with those from high-vacuum deposition processes.

Finally, while the colourized SEM images are attractive, I suggest the original images be added to the SI as the colourizing makes it difficult to observe the details in the SEM images.

True. In the revised version of the Supplementary Information, in the new Fig. S23, we provide all electron micrographs shown in Figs. 1 to 4 of the main paper in not-colored form:

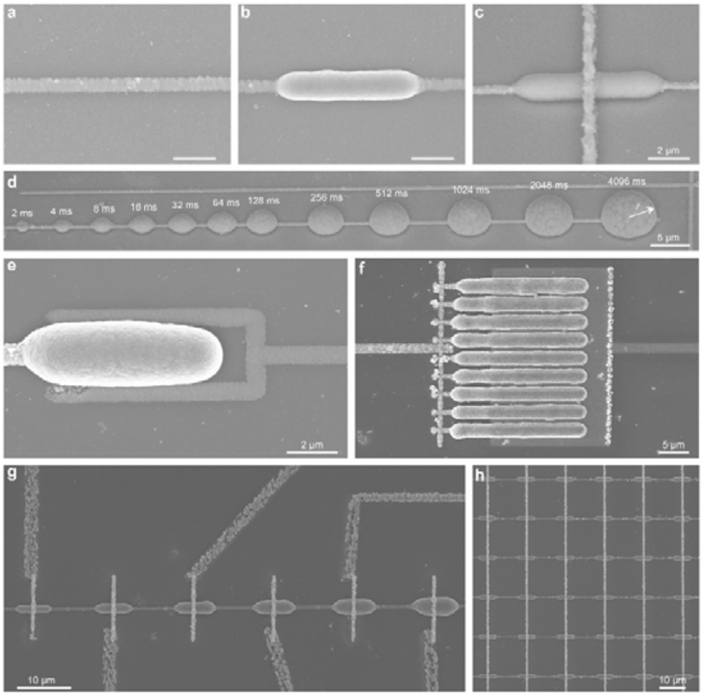

"**Figure S23: Not-colored versions of all electron micrographs of main paper.**

a-c as Fig. 1 d-f, **d**, as Fig. 2a, **c** and **f**, as Fig. 3 a and c, **g**, as Fig. 4a. **h**, as Fig. 5c."

Reviewer #3

The authors present a multi-material laser printing approach for metals and semiconductors with a resolution of approx. 1 µm. Their main invention is an ink for the thermal deposition of ZnO, while the two-photon reduction of metals has been established earlier. Yet, it is this invention that crucially lets the authors print a variety of microeletronic devices. Importantly, the inorganic materials are functional in their as-printed state and do not require a post-print heat treatment. In general, the work will be of significant interest to the field and also a broader audience. The author's methodology is sound and their conclusions are sufficiently supported with data.

We thank the reviewer for this detailed and very positive assessment of our paper. They fully grasp the novelty of our work. We especially thank the reviewer for stating that our "… work will be of significant interest to the field and also a broader audience. …" This statement should be very important for the editor.

I recommend acceptance of the manuscript after major revisions.

Below a few questions and suggestions to the authors.

Questions and comments to the authors:

1. It would be justified to mention the use of multiple inks and the sequential deposition process in the abstract—after reading the abstract, it was not clear to me that different inks were used, and I first thought the different materials were deposited from a single ink. The fact that multiple inks were used is important and should be briefly mentioned. Also, the washing steps between materials should be mentioned in the main text to paint the complete picture of the approach.

We have revised the abstract as follows:

"… Herein, we introduce laser printing with three different inks, for the semiconductor ZnO and the metals Pt and Ag, as a facile process for fabricating printed functional electronic devices with minimum feature sizes below 1 µm. The ZnO printing is based on laser-induced hydrothermal synthesis. Importantly, no sintering of any sort needs to be performed after laser printing for any of the three materials. To demonstrate the versatility of our approach …"

Note that we have at this point also eliminated from the abstract the statement on sintering during the laser-printing process. While we still feel that this statement is true, we have no evidence for this statement (also see below).

The washing step has already been described in the main paper on page 6:

"… The photothermally induced reaction locally converts the ink into polycrystalline ZnO deposited onto the platinum wire, to which it directly forms an electrical contact. After laser printing, the remaining ink is simply washed out, which forms the development step (for further details see Methods). We have successfully …"

Therefore, we feel that no further changes are necessary.

2. The authors don't show any geometry or material that cannot be done with conventional lithography-based processing. Can the authors briefly motivate the use of laser-printing for these kind of devices in the introduction? For the case of 3D architectures the advantage of two-photon lithography is exceptionally clear compared to lithography. For planar, three component electronics with moderate submicrometer resolution, the advantage over lithography is not directly apparent to the reviewer.

In response to reviewer #2, we have already modified the conclusions in this regard. For convenience of reviewer #3, we repeat these changes here:

"… We provided a number of proof-of-principle demonstrations for direct laser printing of functional electronic devices and entire electronic circuits with minimum feature sizes down to <1 µm. A crucial prerequisite for this breakthrough has been the definition of an inorganic semiconductor (ZnO) ink for laser printing via photothermal synthesis. This ink and the associated printing modalities are conceptually distinct from both laser printing of polymers and metals. The advantages of direct laser printing of memristors and circuits based thereupon are the complete additive manufacturability, the possibility of 2D and 3D integration in one process, and the flexibility to design each individual device according to the intended electrical behavior. This especially implies that no high-vacuum deposition and no standard cleanroom lithographic processes are needed for any of the devices presented in this work."

These benefits are probably immediately obvious to anybody working in the field of printed electronics in general. Nevertheless, we now also address this point in the first sentence of the introduction:

"… Throughout the last two decades, many different functional organic[1-3] and inorganic[4-6] electronic devices have been realized by inkjet printing[7-9], laser-induced forward transfer (LIFT)[10,11], or other printing modalities[12-15], rather than by high-vacuum deposition of materials and standard cleanroom lithographic means. Frequently, however, some …"

3. In general, the manuscript would be greatly elevated if the authors could show quantitative analysis of the electronic properties of the printed materials, as these are fundamental for high-performing microelectronics (band gap, conductivity). Could the authors please share conductivity data for printed lines and compare these to bulk conductivity or the conductivity of thin films?

We agree with reviewer #3 in that it would be highly desirable to independently characterize the printed materials, especially the novel ZnO, in terms of their electronic properties. Unfortunately, such characterization of (sub)micron-sized materials is notoriously difficult. Unlike for high-vacuum deposited films, we (and others) cannot simply measure Hall mobility and alike. Therefore, we just cannot provide the information requested by reviewer #3.

The electrical conductivity of Pt or Ag lines has been investigated in the corresponding papers cited in the main text. We have revised the main text as follows:

"… platinum nanoparticles themselves (see Fig. 1a). In this process, light-induced forces onto the metal nanoparticles play a major role for nanoparticles aggregation as well[26]. Due to the large thermal conductivity of the surrounding aqueous ink, the heating can be strongly localized to below a micrometer[28]. The electrical conductivity of laser-printed Pt lines has been characterized[27].

The Ag-"ink" in this work consists of a Ag(I) complex and a citrate reducing agent (also see Methods). Here the photoreaction …"

and

"… Notably, silver is a standard material in inkjet-printed electronics as well as for many types of memristors in general. Polycrystalline silver has a work function of 4.26 eV[34], which complements the much larger work function of platinum of 5.64 eV[35]. The electrical conductivity of laser-printed Ag lines has been characterized[29,30]. Gold can be laser printed along the same lines as well[36,37], but is not used in the present work.

The processes underlying the laser printing of ZnO …"

Along similar lines, the authors mention in-situ laser sintering of the metal lines (Page 3 bottom). It would be helpful to show proof of this sintering in the main text, that is, a high-resolution SEM image of the morphology of these lines (or, if available, a cross-section).

As pointed out above, we have eliminated the statement on in-situ laser sintering from the abstract. We still feel that this statement is correct, but it is conceptually impossible to provide control experiments as the laser not only potentially sinters but also creates the material in the first place. For convenience of reviewer #3, we repeat the modified part of the abstract:

"… Herein, we introduce laser printing with three different inks, for the semiconductor ZnO and the metals Pt and Ag, as a facile process for fabricating printed functional electronic devices with minimum feature sizes below 1 µm. The ZnO printing is based on laser-induced hydrothermal synthesis. Importantly, no sintering of any sort needs to be performed after laser printing for any of the three materials. To demonstrate the versatility of our approach …"

For the case of ZnO, we indeed already show the requested high-resolution SEM image as the inset in Fig. 1(e). The reviewer has possibly overlooked this SEM, which we had put there for exactly that purpose.

We will come back to this SEM and the corresponding text under point 6 of reviewer #3.

4. The authors highlight the fact that their materials are functional without a post-print heat treatment. I strongly agree that this is an important advantage. Nonetheless, I would suggest the authors argue in the introduction why they think the general sintering procedure is a crucial challenge to be avoided, so that the uninformed reader can appreciate this advantage?

Post-sintering is not a scientific challenge, but it is a "pain in the neck". At the end of the introduction we write:

"… Finally, most solution-processable functional materials so far require some sort of sintering process of the involved semiconductors and/or metals at elevated temperatures. This complicates the processing of multilayer devices because caution has to be exerted that the sintering of one material component does not deteriorate any of the other material components involved. …"

We would like to refrain from further dwelling on this point because dwelling on it would mean that we further criticize previous work of others.

5. The authors have missed some of the early work on metal printing by two-photon reduction and cite later work. I think they should merit the original, earlier work. (As a side note, I have extracted these references from a review article on high-resolution metal printing: Hirt, L., Reiser, A., Spolenak, R., Zambelli, T., Hirt, L., Spolenak, R., & Zambelli, T. (2017). Additive Manufacturing of Metal Structures at the Micrometer Scale. Advanced Materials, 29(17).)

a. References to be considered for Ag printing:

i. F. Stellacci, C. Bauer, T. Meyer-Friedrichsen, W. Wenseleers, V. Alain, S. Kuebler, S. Pond, Y. Zhang, S. Marder, J. W. Perry, Adv. Mater. 2002, 14, 194.

ii. T. Tanaka, A. Ishikawa, S. Kawata, Appl. Phys. Lett. 2006, 88, 81107.

iii. Y. Y. Cao, N. Takeyasu, T. Tanaka, X. M. Duan, S. Kawata, Small, 2009, 5, 1144.

iv. A. Ishikawa, T. Tanaka, S. Kawata, Appl. Phys. Lett. 2006, 89, 113102.

b. For two-photon deposition of Au and Cu:

i. W.-E. Lu, Y.-L. Zhang, M.-L. Zheng, Y.-P. Jia, J. Liu, X.-Z. Dong, Z.-S. Zhao, C.-B. Li, Y. Xia, T.-C. Ye, X.-M. Duan, Opt. Mater. Express 2013, 3, 1660.

ii. F. Stellacci, C. Bauer, T. Meyer-Friedrichsen, W. Wenseleers, V. Alain, S. Kuebler, S. Pond, Y. Zhang, S. Marder, J. W. Perry, Adv. Mater. 2002, 14, 194.

We have added three of these references for Ag printing (namely the three highlighted in gray above, in chronological order). In the main text on page 4, these are referenced at:

"… complex followed by reduction of the excited state species. Laser printing of silver has also been discussed many times in the literature[29-32] and is possible with laser-focus speeds up to v=1 cm/s for optimized inks[33]. Laser-printed polycrystalline silver metal lines …"

and in regard to Au we have taken reference b *i*:

"… Polycrystalline silver has a work function of 4.26 eV[31], which complements the much larger work function of platinum of 5.64 eV[32]. The electrical conductivity of laser-printed Ag lines has been characterized[29,30]. Gold can be laser printed along the same lines as well[36,37], but is not used in the present work.

The processes underlying …"

6. Just above Fig 2, in the main text, authors discuss the microstructure of ZnO and mention that the material is composed of ZnO nanoparticles. This terminology suggest an agglomeration of nanoparticles rather than a dense material. Is this the case? The analyses in the SI do not give a definitive answer to this question either.

This point is related to part of point 3 of reviewer #1. We recall that the inset of Fig. 1e shows a high-resolution SEM image of the ZnO surface. There, nanoparticles are clearly visible. Hence, the material is definitely not a single crystal. Nevertheless, we would still call it "dense". These nanoparticles at the laser-printed-ZnO surfaces are also visible in all other electron micrographs shown in the main paper and the Supplementary Information. Beyond that, we cannot say more than that we have shown detailed current-voltage characteristics of many manufactured devices in the main paper as well as in the Supplementary Information.

We come to the nanoparticle size in the next paragraph.

Along the same lines, it would be helpful to the reader if the authors could briefly mention the main conclusions from SI Figs 7-9 in the main text.

We have revised the main text on page 7 as follows:

"… and the Ag printing step are shown in panels d-f of Fig. 1, other structures in Supplementary Figs. 5 and 6. The inset in Fig. 1e shows a magnified high-resolution electron micrograph. From 50 nanoparticles therein, we roughly estimate a mean ZnO nanoparticle size of 27±5 nm. We have performed further detailed characterization of the laser-printed ZnO by scanning-electron energy-dispersive X-ray analysis (see Supplementary Figs. 7 and 8), by high-resolution transmission electron

microscopy, and Raman spectroscopy (see Supplementary Fig. 9) provides evidence that the laser-printed material is actually ZnO.

The lateral and vertical extent of the laser-printed ZnO structures …"

Herein, we have also repeated the changes in regard to the nanoparticle-size measurements that we have made in response to reviewer #1 (see above).

7. For the memristor applications, could the authors briefly mention to the uninformed reader why two different electrode materials (Pt and Ag) are required for this application? Is the use of two different metals crucial or would two Ag electrodes work as well?

Our laser-printed memristors follow the same design as the diodes we present. For the diodes, one obviously needs an asymmetric structure, i.e., they would not work using only a single metal.

More generally, for memristor development, the combination of electrode materials (symmetrical, asymmetrical), the active layer and interface of the active layer between both electrodes, directly influences the resulting device behavior. From our perspective, it should also be possible to fabricate symmetric Ag/ZnO/Ag structured memristors. The review paper discussed under point 8. discusses pros and cons of symmetric and asymmetric memristors (see below).

However, in this work, we intend to utilize an efficient manufacturing workflow for fabricating different microelectronic devices (diodes, memristors, transistors), which can be directly co-integrated in electronic circuit architectures, using the same manufacturing steps (see Fig. 1). In other words, we could probably laser-print Ag-ZnO-Ag memristors. In fact nearly any stack sequence is possible. We have just chosen the sequence Pt-ZnO-Ag that allows for the most efficient integration, in particular in the crossbar array shown in Fig. 5c.

8. For the memristor device, could the authors specifically state how the performance of the laser printed memristors compare to conventional devices? Is it comparable? The authors say their performance is of "high endurance" and "extremely stable", but for the reader from outside the memristor field, the performance is difficult to put into perspective. Please help the reader doing so.

We do not intend to compete with devices made by standard (cleanroom) processing. Our devices are either first-of-a-kind or they can compete in terms of stability and endurance with many reported devices. In more detail, we have changed the main text as follows:

"… Only a few set operations have failed during the 700 switching cycles. This behavior of the fully laser-printed memristor can be compared with other approaches. Compared to solution-processed devices, the endurance in terms of number of switching cycles of our device is better than that of most devices from other reports[41].

We note that the size of the semiconducting ZnO layer of a memristor in crossbar can be flexibly controlled by the laser printing …"

This change implies that our previous more qualitative wording of "high-endurance performance" has been eliminated.

We have also taken out the word "extremely" in:

"… As can be seen from Fig. 4e, both states are stable over the entire measurement period, with no sign of degradation.

To examine the dynamic performance of the device when …"

9. How did the authors calibrate the printed thermocouples?

We have modified the relevant text in the Supplementary Information as follows:

"… Combined with an ambient temperature of 22°C, the measurements performed in air convert to absolute temperatures between 60°C and 100°C, resulting in temperatures between 50°C and 110°C throughout the couple's junctions at a laser power of 10%P_0. For converting the measured thermo-voltages into temperature, we have used the literature value for the Seebeck coefficient of silver relative to platinum of 6.5 µV/K. As usual for macroscopic thermocouples, independent calibration experiments have not been performed. The multiple experiments performed on a single thermocouple …"

10. Could you mention with what range of substrates the process could be compatible other than glass? Does the substrate have to be transparent, or are there any other limitations? Could you deposit the ZnO on a metallic substrate or on an insulating substrate?

In the way we have performed the experiments reported in the paper, the substrate is glass and the substrate needs to be transparent (see, e.g., Figs. 1a and 1b). However, the experiments could likely also be performed in the so-called dip-in mode, for which the substrate need not necessarily be transparent. However, we stress that we have not actually used the dip-in mode. We would like to refrain from too much speculation on things other than what we have specifically done in our paper.

In regard to metallic substrates, ZnO can be deposited on metallic substrates. However, this is not meaningful for the electrical devices presented in our paper as the metallic substrate would short-circuit the devices.

In response to reviewer #2 (see above), we have shortened the conclusions. In particular, we have removed all speculations about possible future directions. This also includes the brief discussion on substrates. For convenience of reviewer #3, we repeat these changes here:

"… We provided a number of proof-of-principle demonstrations for direct laser printing of functional electronic devices and entire electronic circuits with minimum feature sizes down to <1 µm. A crucial prerequisite for this breakthrough has been the definition of an inorganic semiconductor (ZnO) ink for laser printing via photothermal synthesis. This ink and the associated printing modalities are conceptually distinct from both laser printing of polymers and metals. The advantages of direct laser printing of memristors and circuits based thereupon are the complete additive manufacturability, the possibility of 2D and 3D integration in one process, and the flexibility to design each individual device according to the intended electrical behavior. This especially implies that no high-vacuum deposition and no standard cleanroom lithographic processes are needed for any of the devices presented in this work."

11. Out of curiosity: the fact that the authors use different wavelengths for the deposition of the semiconductor and the metals inevitably brings up the question of whether these materials could be selectively deposited from a single, mixed ink? Could the authors comment on this, either just as an answer to me as a reviewer, or, if the authors think it would be important information for the general readers, in the manuscript?

One should perhaps "never say never", but based on our current understanding, it is not possible to define a single ink, from which, using different laser colors, Pt, ZnO, and Ag could be laser-printed. We would like to refrain from writing about things that are supposedly not possible in our manuscript.

12. Two typos: Page 6 (page numbers and line numbers would be helpful), middle. Authors write: "A detailed parameter study has shown that the proper choice of the pH value of the ink is crucial to

avoid resolution of the printed material by the ink itself after the laser exposure (see Supplementary Video 2)." I assume there is a typo, and "resolution" should be "dissolution"?

We have changed this sentence to:

"… A detailed parameter study has shown that the proper choice of the pH value of the ink is crucial to avoid dissolution of the printed material by the ink itself after the laser exposure (see Supplementary Video 2). …"

Experimental section, ZnO ink, first line, "Zno" instead of "ZnO".

Thanks. We have fixed this typo:

"… *ZnO ink*: To obtain a ZnO ink with both a precise …"

Finally, we should sincerely like to thank reviewer #3 once again for their very careful reading of our manuscript. Their detailed points have significantly helped us to improve our manuscript.

REVIEWERS' COMMENTS

Reviewer #1 (Remarks to the Author):

With this revised version, the Authors fulfilled all the concerns I previously raised. The provided replies are convincing and I think the paper is now suitable for publication without the need for further changes.

Reviewer #2 (Remarks to the Author):

Taken as a whole, I am satisfied with the revisions, and find the manuscript publishable. I still have serious doubts regarding the ultimate impact, but this is due to my broader doubts about the potential of the field of printed electronics, and not technical concerns related to this specific manuscript.

Reviewer #3 (Remarks to the Author):

I thank the authors for their review. My comments have been adequately addressed and I recommend accepting the article for publication in its current form.

In response to my comment #3, which inquired about materials properties, the authors added references to previous characterization of conductivity of printed metal lines. I would certainly have preferred a statement of the actual values of conductivities and their comparison to bulk values in the author's manuscript, so that readers can appreciate these properties without looking up a reference first. But this is a personal preference.

Point by Point Response

Manuscript number: NCOMMS-21-16739B

Title: Development of the oral resistome during the first decade of life

Please find below our point-by-point response to the reviewers' comments. The reviewers' comments are *Italicised* and our responses are provided sequentially following reviewers' comments. Line numbers refer to the updated, track change version of the paper.

Reviewer #1 (Remarks to the Author):

The authors have addressed efficiently the comments.

Reply: Thank you.

Reviewer #2 (Remarks to the Author):

In this rebuttal, the authors address all concerns brought up in our previous review. The adjustments made to help with redundancies in the text and sentence structure were very helpful and sufficient. We also appreciate the authors undertaking the effort to examine the changes in functional pathways over time and are excited to see that the authors found correlations between macrolide resistance genes and antibiotic detoxification. However, the new figure displaying these findings is difficult to read and some adjustments could help with visibility.

Reply: We agree and have updated Figure 3. Please see our detailed response below, under Minor concerns, point 4.

Further, the authors claim to have found significant correlations between early feeding patterns and resistome phenotype. The p-values used in this analysis need to be corrected for multiple comparisons.

Reply: Thank you for picking this up. We have now performed multiple test correction and the findings have remained the same. Please see our detailed response, under Major concerns, point 2.

While we appreciate the additional analysis and structure the authors have provided for this publication, there are still a number of concerns that need to be addressed.

Major concerns
1. 309-313: Why would the environment stabilize at T2 but become more variable again at T3? There is no explanation as to why this would occur.

Reply: We agree that the reasoning for the change in heritability over time was not clearly justified. In brief, we suspect that we may have 'missed' the effect of heritability at Time point 1 (T1) due to insufficient sample size and hence power. Heritability was calculated on 28 twin pairs at T1 compared to 38 at T2 and 43 at T3. To reliably estimate heritability of complex phenotypes, such as the resistome, generally requires large numbers of twin pairs. For example, in a study of the effect of heritability on the gut microbiome using metagenomic data, 127 adult twin pairs were assessed[1]. Underestimates of heritability due to insufficient sample size have been raised as a concern for twin studies using genomic data[2].

In our study, we suspect the level of heritability at T1 is higher, potentially more at the level of T2 and then declined between T2 and T3. The lower level of additive genetic effects and increased environmental influence observed at T3 (~8 years) compared to T2 (1 – 2 years) is potentially due to increasingly diverse environmental exposures of school aged compared to younger children. To clarify this issue, we have re-worded the Discussion (updated line numbers 312 – 322), explaining that while we can describe the pattern of heritability from T2 onwards, we have concerns about the T1 additive genetic effect values due to potentially insufficient sample size.

2. 182-185: The p-values mentioned are a few values from a large number of comparisons done and are not adjusted for multiple hypothesis testing. These corrections need to be made before they can be deemed significant.

Reply: For the assessment of environmental factor influences (feeding, SES, antibiotics, etc.) on the resistome we have now performed multiple test correction using the Benjamini & Hochberg method (updated line numbers 197 – 200 and line numbers 446 – 448). Please note, we applied the p-value adjustment method to specific ARGs tested and not to the overall ARG diversity metric, given this is a single metric and not the same data type as the abundance of specific ARGs. Table S12.2, S12.3 and S12.4 now report the multiple test corrected p-values. Following multiple test correction, a significant relationship was still found at T2 between the abundance of ARGs and breast compared to bottle feeding.

3. line 240 onwards: The association between caries severity and biofilm related pathways remains unclear. Either remove this mention of "caries" from the hypothesis or elaborate on caries-associated findings. It is also seemingly contradictory to expect more severe caries to have more diverse resistomes, in light of the Result that CF resistomes were higher in richness than CA.

Reply: We agree that the relationship between caries, biofilm pathways and the resistome was unclear. As suggested, we have removed mention of caries from the hypothesis, focusing on the relationship between diversity of ARGs and abundance of biofilm promoting pathways. The updated description of these results (line numbers 142 – 152) and their discussion (line numbers 282 – 288) has been moved from the caries part of the paper to the section on the relationship between functional pathways and the resistome.

Minor concerns:
1. Line 134: "and ARGs, overtime, we used" -> "and ARGs over time, we used"

Reply: Updated line number 134: edited as advised.

2. 142-144: The resistome had the most interactions with species as opposed to what? What do the numbers 44 and 55 signify?

Reply: This sentence has been revised and now reads "The DIABLO correlation analysis revealed that overall the resistome had more potential interactions with species than functional pathways." (Updated line numbers 153 – 154).

3. 150: spell out insertion sequences (IS) at first instance

Reply: Updated line numbers 161 – 162: edited as advised.

4. Figure 3 is difficult to interpret. Something needs to be done to make the lines differentiable, either removing the species section of the table, or adjusting thickness by correlation, etc. Some of the text labels are cut off along the top as well. "Expression" levels are also not clearly legible (what is the unit/axis?). may benefit from removing that layer of data from plot.

Reply: We agree that Figure 3 was too complicated. To improve visibility, we have made the following changes:

A. Increased correlation cut-off from 0.7 to 0.75: This has reduced the number of correlations, while preserving the pattern from the original plot. Correlation values from the DIABLO analysis provided in Table S5 have been updated to only include those over 0.75.

B. Changed connecting lines colour: To improve clarity, positive correlation values are now blue and negative black, making it easier to differentiate lines.

C. Highlighted ARG-function correlation of interest: We have placed yellow boxes around the text labels for MacA and mycothiol biosynthesis.

D. Removed expression data layer: In short, the expression level information was the vector loading values from the discriminatory analysis performed as part of DIABLO per data block. This showed which ARGs, species and functional pathways most contributed to the clustering by time point. We have presented this information as vector/plot loadings in the supplementary material (updated Figure S4).

E. Text labels are all in full, with no text cut-off.

5. Figure 4: “Bacteroidetes” text is flipped.

Reply: Figure 4 has been edited as advised.

6. 287: Maybe change to say that brushing/extraction can cause bacteremia, rather than does cause. This line also needs a citation.

Reply: Edited as advised with a new citation[3]. Updated line number 293 – 294 now reads “Dental interventions like daily oral activities e.g., tooth brushing, may cause bacteraemia.”

7. 289: “causing , infective endocarditis, and” -> “causing infective endocarditis and ventilation”

Reply: Edited as advised, comma has been removed in line 296.

8. 308-309: is there any previous literature discussing heritability being underestimated at earlier timepoints?

Reply: Please see response to Major concerns, point 1.

9. Table S4: make sure “sex” and “gender” are used distinctively

Reply: Title of Table S4 have been edited and now reads:

Table S4.1: Relationship between resistome composition (Shannon ARG) and microbiome/species composition (Shannon SP). Age and sex were not adjusted for because neither were found to have a significant effect.

Table S4.2: Relationship between resistome composition (Shannon ARG) and functional pathways (Shannon Function). Age and sex were not adjusted for because neither were found to have a significant effect.

References

1. Xie H*, et al.* Shotgun metagenomics of 250 adult twins reveals genetic and environmental impacts on the gut microbiome. *Cell systems* **3**, 572-584. e573 (2016).

2. Mayhew AJ, Meyre D. Assessing the Heritability of Complex Traits in Humans: Methodological Challenges and Opportunities. *Curr Genomics* **18**, 332-340 (2017).

3. Tomás I, Diz P, Tobías A, Scully C, Donos N. Periodontal health status and bacteraemia from daily oral activities: systematic review/meta-analysis. *J Clin Periodontol* **39**, 213-228 (2012).
